# Non-gonadal somatic piRNA pathways ensure sexual differentiation, larval growth, and wing development in silkworms

**DOI:** 10.1371/journal.pgen.1010912

**Published:** 2023-09-21

**Authors:** Takashi Kiuchi, Keisuke Shoji, Natsuko Izumi, Yukihide Tomari, Susumu Katsuma

**Affiliations:** 1 Department of Agricultural and Environmental Biology, Graduate School of Agricultural and Life Sciences, The University of Tokyo, Yayoi 1-1-1, Bunkyo-ku, Tokyo, Japan; 2 Institute for Quantitative Biosciences, The University of Tokyo, Yayoi 1-1-1, Bunkyo-ku, Tokyo, Japan; 3 Department of Computational Biology and Medical Sciences, Graduate School of Frontier Sciences, The University of Tokyo, Bunkyo-ku, Tokyo, Japan; University of Kentucky, UNITED STATES

## Abstract

PIWI-interacting RNAs (piRNAs) guide PIWI proteins to target transposons in germline cells, thereby suppressing transposon activity to preserve genome integrity in metazoans’ gonadal tissues. Piwi, one of three *Drosophila* PIWI proteins, is expressed in the nucleus and suppresses transposon activity by forming heterochromatin in an RNA cleavage-independent manner. Recently, Piwi was reported to control cell metabolism in *Drosophila* fat body, providing an example of piRNAs acting in non-gonadal somatic tissues. However, mutant flies of the other two PIWI proteins, Aubergine (Aub) and Argonaute3 (Ago3), show no apparent phenotype except for infertility, blurring the importance of the piRNA pathway in non-gonadal somatic tissues. The silkworm, *Bombyx mori*, possesses two PIWI proteins, Siwi (Aub homolog) and BmAgo3 (Ago3 homolog), whereas *B*. *mori* does not have a Piwi homolog. Siwi and BmAgo3 are mainly expressed in gonadal tissues and play a role in repressing transposon activity by cleaving transposon RNA in the cytoplasm. Here, we generated *Siwi* and *BmAgo3* loss-of-function mutants of *B*. *mori* and found that they both showed delayed larval growth and failed to become adult moths. They also exhibited defects in wing development and sexual differentiation. Transcriptome analysis revealed that loss of somatic piRNA biogenesis pathways results in abnormal expression of not only transposons but also host genes, presumably causing severe growth defects. Our results highlight the roles of non-gonadal somatic piRNAs in *B*. *mori* development.

## Author summary

In animal germ cells, PIWI proteins and piRNAs play an important role in the defense system against selfish elements such as transposons. The fruit fly *Drosophila* possesses two PIWI proteins, Aub and Ago3, both of which are required for piRNA production and transposon repression in the cytoplasm of germ cells. However, their roles in somatic cells are unclear. Using a lepidopteran model insect, the silkworm *Bombyx mori*, we established Siwi (Aub homolog) and BmAgo3 (Ago3 homolog) germline knockout mutants and characterized non-gonadal somatic piRNA functions in *B*. *mori* development. We found that somatic piRNAs are utilized to suppress transposon activity and maintain normal gene expression in several somatic tissues. Unlike *Drosophila*, disruption of somatic piRNA pathways severely inhibited normal development in somatic tissues of *B*. *mori*: both mutants grew slowly, did not develop to adult moths, and exhibited defects in wing development and sexual differentiation. This study is the first to generate germline knockout insects of PIWI genes in non-*Drosophila* insects and expands the knowledge on the ancestral or universal functions of piRNAs in arthropods.

## Introduction

In animal germline cells, PIWI-clade proteins and PIWI-interacting RNAs (piRNAs) play a major role in the defense against selfish elements and viral transcripts. piRNAs are a class of small RNAs, approximately 24–30 nucleotide (nt)-long; most produced from transposons and other selfish elements. piRNA biogenesis begins with long single-stranded precursors mainly transcribed from piRNA clusters [1–6]. PIWI proteins have piRNA-guided RNA cleavage activity, called slicer. The PIWI–piRNA complex cleaves not only target transposon RNAs but also precursor RNAs for piRNAs [7,8], producing pre-pre-piRNAs [5,6]. These pre-pre-piRNAs are loaded into PIWI proteins and cleaved by the endonuclease Zucchini (Zuc) or a PIWI–piRNA complex [9–12]. PIWI-bound 5′ cleavage fragments, called pre-piRNAs, are 3′ trimmed by the exoribonuclease Trimmer in the silkworm, *Bombyx mori*, or PNLDC1 in mice [13–17]. In the fruit fly, *Drosophila melanogaster*, the 3′-end of pre-piRNAs can be either directly defined by Zuc cleavage [9,10] or slightly trimmed by Nibbler [11]. The 3′-end of pre-piRNAs is 2’-*O*-methylated by the methyltransferase Hen1 [13,18–21].

A subset of PIWI proteins are preferentially loaded with pre-piRNAs with 5′ U (1U) [3,22–24]. The PIWI-1U piRNA complex cleaves their complementary targets between positions 10 and 11 from the 5′-end of guide piRNAs. The cleaved 3′ RNA fragments are then incorporated into another PIWI protein and processed into mature piRNAs with adenine at position 10 (10A), which precisely overlaps with 1U piRNAs by 10 nt and constitute the so-called “ping-pong” signature [3,22–24]. 10A piRNAs can produce new 1U piRNAs by cleaving their complementary target RNAs. This ping-pong cycle is broadly conserved among animals like flies, mice, zebrafish, sponges, and *B*. *mori* [3,24–27].

*Drosophila* possesses three PIWI proteins, Piwi, Aubergine (Aub), and Argonaute3 (Ago3), all expressed in the male and female gonads [3,22,28–30]. Piwi/Aub duplication occurred at the base of the Brachycera, generating the Piwi and Aub subclades [31]. Piwi is localized in the nucleus of germ cells and ovarian somatic follicle cells and represses transposon activity by forming heterochromatin in a cleavage-independent manner [30,32]. On the other hand, Aub and Ago3 repress transposon activity by cleaving transposon RNA in the cytoplasm of germ cells [3,22]. Loss-of-function mutations in these three genes result in sterility or semi-sterility in both sexes [29,33–35]. Recently, Piwi was reported to be expressed in the adult fly fat body and to control cell metabolism and normal lifespan [36]. Sequencing of small RNA populations from the female head and thorax (without ovaries) of the Asian tiger mosquito, *Aedes albopictus*, infected with chikungunya virus provided evidence for the production of virus-derived piRNA-like small RNAs via the ping-pong cycle in the soma [37]. Moreover, in the migratory locust, *Locusta migratoria*, the Piwi-like protein Piwi1 is expressed in the brain and involved in food intake by regulating neuropeptide *NPF1* expression [38]. In the tobacco hawkmoth, *Manduca sexta*, both *Aub* and *Ago3* genes are expressed in the intersegmental muscles (ISMs) [39]. Both genic and transposon-derived piRNAs are expressed in the ISMs and have the ping-pong signature. In the whitefly, *Bemisia tabaci*, four PIWI proteins are encoded in the genome, two of which are expressed in the guts, salivary glands, and whole body [40]. The piRNAs found in the whole body of the whitefly possess the ping-pong signature. The evidence for the ping-pong cycle of transposon- and endogenous viral element-derived piRNAs in somatic tissues was also reported in the Asian citrus psyllid, *Diaphorina citri* [41]. Lewis *et al* [42] showed that transposon-derived piRNAs with a ping-pong signature are detected not only in germ cells but also in somatic cells throughout arthropods. These reports suggest the importance of the somatic ping-pong pathway and transposon silencing, in other words, the roles of Aub and Ago3 in non-gonadal somatic tissues, but direct evidence is lacking.

The piRNA pathway in *B*. *mori* has been mainly characterized in the ovary-derived cell line BmN-4 [24]. The *B*. *mori* ping-pong cycle requires two PIWI proteins Siwi (*B*. *mori* functional Aub orthologue) and BmAgo3 (*B*. *mori* functional Ago3 orthologue), whereas *B*. *mori* does not have a Piwi homolog [43]. The Siwi–1U piRNA complex cleaves its complementary targets, and cleaved 3′ fragments are incorporated into BmAgo3. By cleaving its target RNAs, BmAgo3–10A piRNA complex produces a 1U piRNA precursor. In addition to Siwi and BmAgo3, several other factors involved in *B*. *mori* piRNA biogenesis, including Trimmer, *B*. *mori* Vasa (BmVasa), and *B*. *mori* Zuc (BmZuc), have been identified and characterized [12–14,44,45]. Recently, *Siwi* and *BmAgo3* knockout (KO) strains have been established by the transgenic CRISPR/Cas9 method [46–49]. By characterizing the F_1_ progenies obtained by crossing the *IE1*-*Cas9* and *U6*-*Siwi* or *U6*-*BmAgo3 sgRNA* transgenic lines, *Siwi* was, as expected, demonstrated to be crucial for female germ cell development and transposon silencing, whereas a *BmAgo3* KO affects only oogenesis [46,50]. Moreover, *Siwi* was required for feminization, reflecting the unique piRNA-dependent sex-determination system in *B*. *mori* [46,51]. However, these experiments were conducted using inducible KO lines, and the efficiency of KO depends on the promoter activity and varies in different tissues. Therefore, the complete “loss-of-function” phenotypes of *Siwi* and *BmAgo3* have not been evaluated. Moreover, the properties of gonadal and somatic piRNAs have not been compared between wild-type (WT) and KO lines.

In this study, we established and characterized *Siwi* and *BmAgo3* germline KO mutants and found the role of the non-gonadal somatic piRNA pathway in *B*. *mori* development, including sexual differentiation. The somatic piRNAs generated via ping-pong cycle suppress transposon activity and maintain normal gene expression.

## Results

### Phenotypic characterization of *BmAgo3* and *Siwi* KO mutants

**Fig 1:**
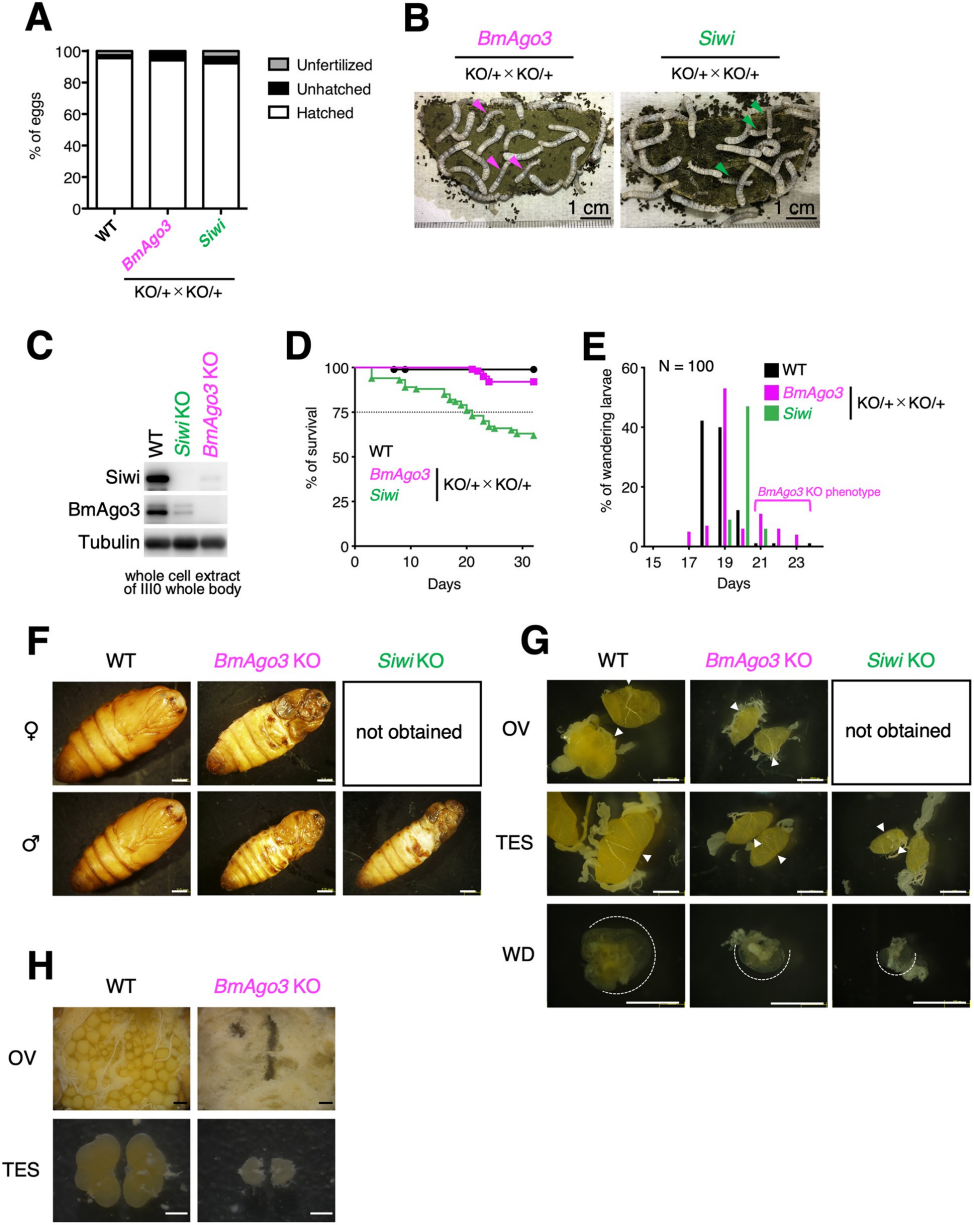
Phenotypic characterization of *BmAgo3* and *Siwi* KO mutants. (A) Hatching rate in crosses between *BmAgo3* or *Siwi* heterozygous KO mutants. To obtain homozygous KO mutants, heterozygous male and female KO individuals were crossed. The average percentages of hatched, unhatched, and unfertilized eggs from 3 (WT and *BmAgo3*) or 4 (*Siwi*) batches are indicated by white, black, and gray bars, respectively. The crossing patterns are indicated in the figure. (B) Developmental delay in *BmAgo3* and *Siwi* KO mutant larvae. Third instar larvae obtained from the parents carrying heterozygous mutations were photographed. Developmentally delayed larvae are indicated by arrowheads. Photographs of all larvae are shown in S2 Fig. (C) Detection of PIWI proteins. Whole cell extracts from the whole body of a day 0 third instar (III0) larva were separated by SDS-PAGE and immunoblotted with anti-BmAgo3, Siwi, and Tubulin antibodies. Developmentally delayed individuals were used as *BmAgo3* and *Siwi* KO. (D) Survival curves of larvae obtained by crosses between *BmAgo3* or *Siwi* heterozygous KO mutants. One hundred newly hatched larvae were reared using an artificial diet and the number of dead larvae was counted every day. (E) Timing of larval wandering in *BmAgo3* and *Siwi* KO mutants. The larval wandering behavior that precedes pupation was delayed in *BmAgo3* homozygous mutants by a few days. Pupae of *Siwi* homozygous mutants were not obtained in this experiment. The number indicates the sample size. (F) Phenotypes of *BmAgo3* and *Siwi* KO mutant pupae. Impaired head and leg morphologies in both homozygous mutants. Female pupae of *Siwi* KO mutants were not obtained. Scale bars, 2 mm. (G) Abnormalities in internal tissues in *BmAgo3* and *Siwi* KO mutant larvae. Ovaries (OV), testes (TES), and wing discs (WD) were dissected from day 4 fifth instar larvae. The positions connected to the duct are indicated by arrowheads. The margin of the wing disc is indicated by a dotted curve. Scale bars, 1 mm. (H) Developmental defect of gonads in *BmAgo3* KO mutant pupae. Day 4 female pupae were dissected and the inside of the abdomen was observed under a microscope. No ovarian eggs were found in *BmAgo3* KO mutant pupae. Testes were removed from day 4 male pupae and observed in phosphate-buffered saline under a microscope. Scale bars, 1 mm.

To understand the role of *BmAgo3* and *Siwi* in *B*. *mori*, we generated *BmAgo3* and *Siwi* KO mutants through CRISPR/Cas9-mediated genome editing. We obtained three *BmAgo3* KO mutants that contained a 2 base pair (bp) deletion, 3 bp insertion and 1 bp deletion, and a 31 bp deletion, respectively, all possibly encoding incomplete BmAgo3 proteins without the MID and PIWI domains (S1A Fig). We also obtained a *Siwi* KO mutant with a 29 bp deletion around the target site, resulting in an incomplete Siwi protein lacking part of the PAZ domain, and the full MID and PIWI domains (S1B Fig). To obtain homozygous KO mutants, we crossed heterozygous male and female KO. All three *BmAgo3* homozygous KO mutants and the *Siwi* homozygous KO mutant showed similar phenotypes. Despite the normal hatchability of both crosses (Fig 1A), about a quarter of larvae exhibited developmental delay at the larval stage (Figs 1B and S2). Genotyping revealed that all delayed larvae had homozygous KO alleles (S1 Table). Western blot analysis revealed that third instar larvae with developmental defects in *BmAgo3* KO crosses and *Siwi* KO crosses did not express BmAgo3 protein and Siwi protein, respectively (Fig 1C). Moreover, we noticed that the amount of Siwi protein was heavily reduced in *BmAgo3* KO larvae, and *vice versa*. Loss of BmAgo3 protein resulted in piRNA depletion (see following section). Judging from the previous studies [52], we speculated that small RNA-free Siwi and BmAgo3 proteins are unstable, presumably resulting in the decrease in the Siwi (BmAgo3) protein in the *BmAgo3* (*Siwi*) KO larvae. Most *BmAgo3* KO larvae with developmental defects entered the wandering stage, started to spin a cocoon, and eventually became pupa (Fig 1D and 1E). On the other hand, delayed larvae in *Siwi* KO crosses gradually died during the larval stage (Fig 1D); finally, no *Siwi* homozygous KO pupae were observed (Fig 1D and 1E). In the maintenance of the *Siwi* KO mutant (where heterozygous individuals were crossed at each generation to maintain the mutation), male pupae with homozygous KO genotype were occasionally obtained. In both KO mutants, pupal weight was significantly decreased (S3A Fig) and morphological abnormalities in the wings were observed (Figs 1F and S3B). Despite the similar pupal morphological abnormalities observed in both KO mutants, *Siwi* KO resulted in more severe defects than *BmAgo3* KO, as head and leg morphologies were also greatly impaired in *Siwi* KO pupae (S3B Fig). On the other hand, the external morphology of the pupal genitalia was normal in both KO mutants (S3B Fig). All *BmAgo3* and *Siwi* KO pupae failed to emerge and died. We dissected and examined the gonads and wing discs of day 4 fifth instar larvae. As shown in Fig 1G, both ovaries and testes in *BmAgo3* KO larvae were smaller compared than in WT larvae. A similar phenotype was observed in the testes of *Siwi* KO larvae (Fig 1G). Wing disc size was also reduced in *BmAgo3* and *Siwi* KO (Fig 1G). We also dissected and observed gonads of day 4 pupae. No ovarian eggs were observed in *BmAgo3* KO female pupae, and developmental defects in testes occurred in *BmAgo3* KO male pupae (Fig 1H).

### Characterization of somatic piRNAs in *B*. *mori* fat body and wing discs

**Fig 2:**
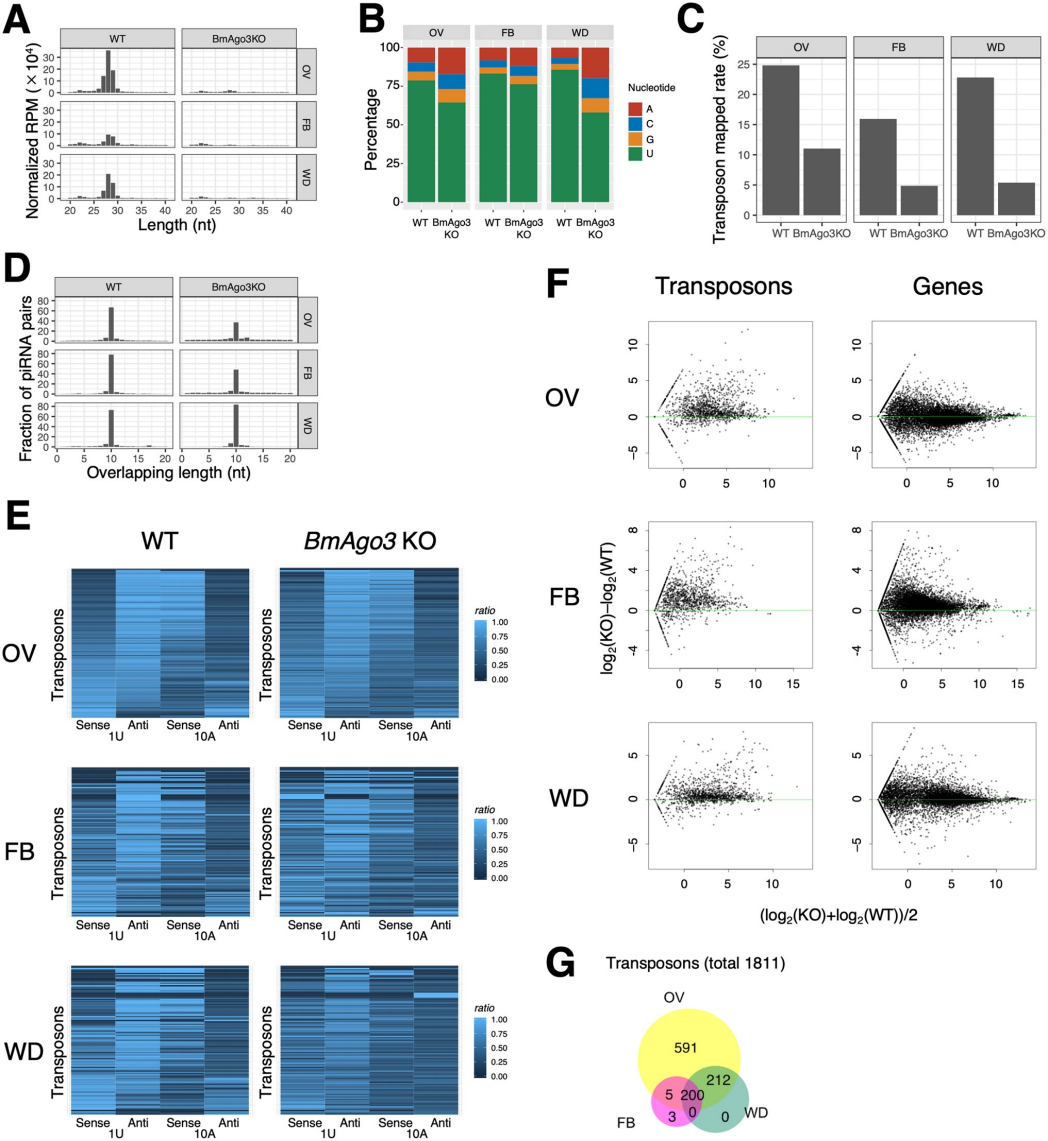
Characterization of *Bombyx* somatic piRNAs. (A) The length distribution of small RNAs in female tissues from fifth instar larvae. The small RNA libraries of WT and *BmAgo3* KO were prepared from 3–4 and 4–8 fifth instar larvae, respectively. Small RNAs around 27 nucleotides long in the ovaries (OV), fat body (FB), and wing discs (WD) disappeared in *BmAgo3* KO. The y-axis indicates normalized reads per million (RPM). (B) First base bias of small RNAs of around 27 nucleotides in length. The percentages of the first nucleotides of the small RNAs shown in (A) were calculated. (C) Mapping rate of each tissue small RNA library to transposons. (D) Investigation of ping-pong signature. piRNAs amplified by ping-pong have overlapping pairs of 10 bases. (E) piRNA orientation and 1U/10A strand bias for each transposon. For each transposon, the 1U bias and 10A bias for each strand were sorted by the difference between sense and antisense percentages of 1U piRNAs in WT ovaries. (F) MAplot for mRNA expression levels using transposons and silkworm gene models. Total RNA used for RNA-seq analysis was the same as that used for small RNA-seq analysis. The transcripts per million (TPM) of female tissues from WT and BmAgo3 KO were used to calculate the difference in M:log_2_(TPM) and the mean value of A:log_2_(TPM). The axes show: A (x-axis) = (log_2_(TPM in *BmAgo3* KO) + log_2_(TPM in WT))/2. M (y-axis) = log_2_(TPM in *BmAgo3* KO)–log_2_(TPM in WT). (G) Venn diagram showing the inclusion relationship of transposons producing more than a certain amount of piRNA (normalized RPM > 4). piRNAs are produced from 1011 transposons out of 1811 *B*. *mori* transposons examined. Ovarian piRNAs are produced from 1008 transposons, of which ovary-specific piRNAs are produced from 591 transposons. Non-gonadal somatic piRNAs are produced from 412 and 208 transposons in wing discs and fat body, respectively. Common piRNAs in ovary and wing discs are produced from 412 transposons. Common piRNAs in ovary and fat body are produced from 205 transposons. Common piRNAs in the three tissues are produced from 200 transposons. Fat body-specific piRNAs are produced from 3 transposons.

To characterize in detail the phenotypic abnormalities of *BmAgo3* KO mutants, we performed small RNA sequencing of ovaries, fat bodies, and wing discs from WT and *BmAgo3* KO fifth instar female larvae. Fifth instar female larvae of *Siwi* KO were not obtained (Fig 1D). We identified abundant 26–30 nt-long small RNAs with 5′ uridine (1U) in all tissues examined (Fig 2A and 2B), 15%–25% of which were mapped to transposons (Fig 2C). In contrast, small RNAs were mostly absent in all tissues of *BmAgo3* KO larvae (Fig 2A). These results strongly suggest that these small RNAs are piRNAs, and that not only germ cells but also somatic tissues produce piRNAs in silkworms. Approximately 65%–75% sense and antisense piRNA pairs overlapped precisely by 10 nt from their 5′ ends in all WT tissues (Fig 2D). We then investigated the strand bias of the mapped 1U and 10A piRNAs for each transposon. We found that 1U and 10A piRNAs were mostly mapped to the opposite strands in fat bodies and wing discs as well as in ovaries (Fig 2E). These findings demonstrate that piRNA production by the ping-pong cycle operates in female gonad and non-gonadal somatic tissues of *B*. *mori*. Ping-pong signatures were observed in the remaining piRNAs of *BmAgo3* KO mutants (Fig 2D and 2E), suggesting the possibility of Siwi:Siwi homotypic ping-pong similar to Aub:Aub homotypic ping-pong in *ago3* mutant flies [53]. Furthermore, RNA-seq analyses revealed globally enhanced expression levels of transposon mRNAs in all three tissues of *BmAgo3* KO larvae (Fig 2F). In *Drosophila*, germline and somatic piRNAs are produced via the ping-pong-dependent and ping-pong-independent pathways, respectively, and these piRNAs can be distinctly classified. However, our results strongly suggest that gonadal and somatic piRNAs cannot be distinguished in *B*. *mori*, and that piRNA amplification and transposon silencing are governed by the ping-pong cycle in both germline and somatic tissues. We also found more diverse transposon species for ovarian piRNAs than those of fat body and wing disc piRNAs, indicating that somatic piRNAs are produced from a relatively limited number of transposon types and most somatic piRNAs are produced from the same locus as gonadal piRNAs (Fig 2G).

To show that the ping-pong cycle is active in *B*. *mori* somatic tissues, we decided to focus on a piRNA enriched in the fat body. This piRNA is produced from a single locus of *storage protein 1* (*SP1*) (*KWMTBOMO13992*) mRNA and accompanies a putative ping-pong partner piRNA produced from *KWMTBOMO03023* mRNA (transposon), the 2–18 nt of which are complementary to *SP1* (S4A and S4B Fig). We previously showed that base-pairing of the 2–18 nt at the 5′-end of the piRNA target sequence is sufficient for the PIWI protein to cleave the target mRNA in *B*. *mori* [54], suggesting that this ping-pong partner is active in the fat body. The *SP1* piRNA was detected almost specifically in the fat body, although its partner piRNA was detectable in all three tissues (S4C and S4D Fig). This is mainly because of the higher expression of *SP1* mRNA in the fat body than in ovaries or wing discs (S4E Fig). On the other hand, *SP1* piRNA is unlikely to recognize *KWMTBOMO03023* mRNA, which is similarly expressed in fat body, ovary, and wing disc (S4F Fig), because only 1–9 nt of *SP1* piRNA are complementary to *KWMTBOMO03023* mRNA (S4B Fig). In addition, no sequences similar to *SP1* piRNA are found in the silkworm genome other than *SP1*. Accordingly, this piRNA partner is not canonical; *SP1* mRNA can be recognized by an antisense piRNA derived from a different locus in trans, whereas *SP1*-derived sense piRNA does not involve antisense piRNA production.

In addition, we searched for genes (other than transposons) to which both sense and antisense piRNAs were sufficiently mapped (RPM > 10) and whose expressions were more than 2-fold up-regulated in at least one of the three tissues in *BmAgo3* KO larvae compared to WT (S2 Table). Ping-pong signatures were observed between sense and antisense genic piRNAs in fat body and wing discs, suggesting that piRNA-mediated gene silencing occurs in non-gonadal somatic tissues as well as gonadal tissues.

### Effects of *BmAgo3* and *Siwi* KO on sex-determining genes

**Fig 3:**
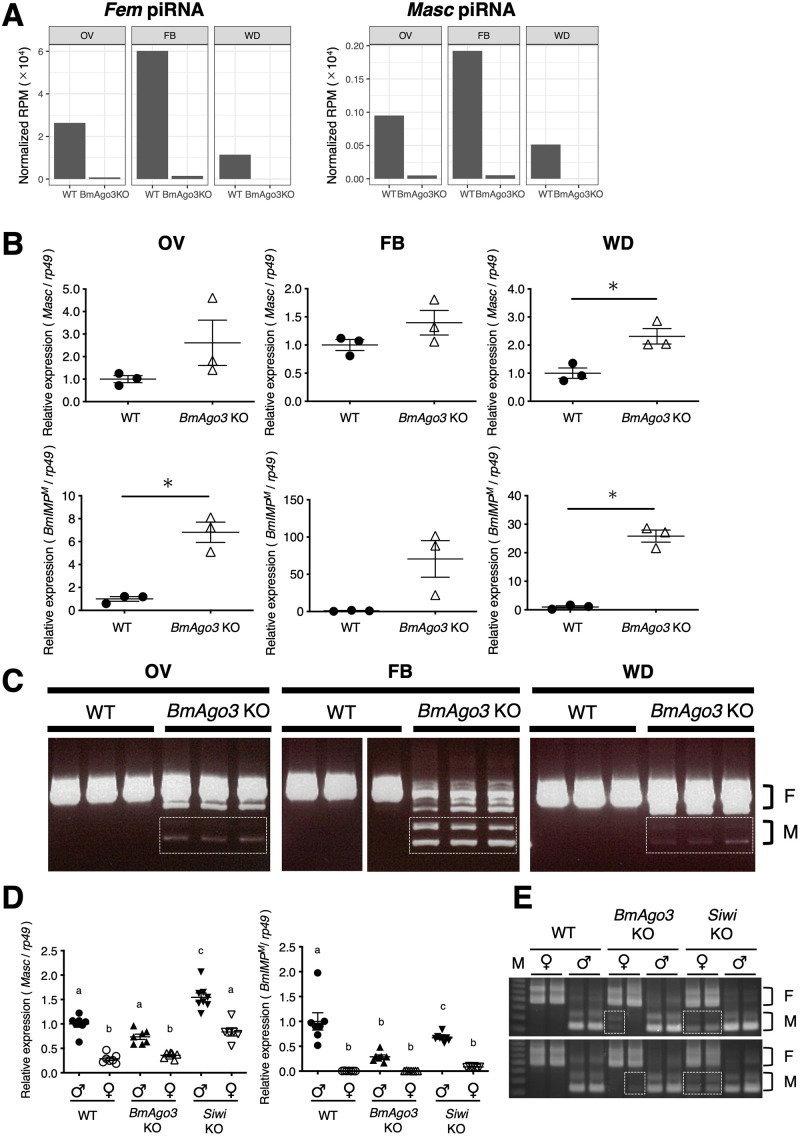
Effects of *BmAgo3* and *Siwi* KO on sex-determining genes. (A) Depletion of *Fem* and *Masc* piRNAs in female tissues of *BmAgo3* KO mutants. Normalized RPM of *Fem* and *Masc* piRNAs in small RNA libraries of the ovaries (OV), fat body (FB), and wing discs (WD) were calculated. The small RNA libraries of WT and *BmAgo3* KO were prepared from tissues of 3–4 and 4–8 fifth instar female larvae, respectively. (B) Increased mRNA levels of masculinizing genes in female tissues of *BmAgo3* KO mutants. *Masc* and *BmIMP*^*M*^ expression levels were examined by RT-qPCR. The relative mRNA levels (wild-type female = 1) were normalized to those of *rp49*. Bars indicate means ± SE of three tissues. Unpaired *t* test with Welch’s correction (*p < 0.05). (C) Representative splicing pattern of *Bmdsx* in female tissues of *BmAgo3* KO mutants. *Bmdsx* splicing was examined by RT-PCR. F and M indicate female- and male-type *Bmdsx* splicing, respectively. Male-type *Bmdsx* splicing was observed in the female tissues of *BmAgo3* KO mutant larvae (dotted boxes). (D) Expression levels of masculinizing genes in *BmAgo3* and *Siwi* KO mutant larvae. *Masc* and *BmIMP*^*M*^ expression levels in the whole body of a day 0 third instar larva examined by RT-qPCR. The relative mRNA levels (wild-type male = 1) were normalized to those of *rp49*. Larval sex was determined by RT-qPCR analysis using a W-specific marker, *Fem*. Bars indicate means ± SE of 6–8 larvae. One-way analyses of variance (ANOVA) were performed with post hoc Tukey–Kramer test (*p* < 0.05). Different letters indicate significant differences between groups. (E) Representative splicing pattern of *Bmdsx* in *BmAgo3* and *Siwi* KO mutant larvae. *Bmdsx* splicing in the whole body of a day 0 third instar larva examined by RT-PCR. F and M indicate female- and male-type splicing of *Bmdsx*, respectively. Male-type *Bmdsx* splicing was observed in female larvae of *BmAgo3* and *Siwi* KO mutants (dotted boxes).

In *B*. *mori*, a single female-specific piRNA (*Fem* piRNA) derived from the W chromosome is essential for female sex determination [51,55]. The Siwi–*Fem* piRNA complex targets the masculinizing gene *Masc*. The cleaved 3′ fragment of *Masc* mRNA produces *Masc*-derived piRNA, which is partially complementary to *Fem* piRNA and binds to BmAgo3. The BmAgo3-*Masc* piRNA complex in turn cleaves *Fem* RNA and produces *Fem* piRNA [51]. As shown in Fig 3A, *Fem* and *Masc* piRNAs were detected not only in the ovary, but also in somatic tissues, i.e., fat body and wing discs. Moreover, both piRNAs were almost absent in *BmAgo3* KO tissues (Fig 3A), raising the possibility that loss of BmAgo3 impacts the sex determination cascade. Indeed, in *BmAgo3* KO female larvae (fifth instar), both *Masc* mRNA levels and male-type variant of *B*. *mori IGF-II mRNA-binding protein* (*BmIMP*^*M*^) showed an increasing trend in the ovary (*Masc*: *p* = 0.2541, *BmIMP*^*M*^: *p* = 0.0183), fat body (*Masc*: *p* = 0.2401, *BmIMP*^*M*^: *p* = 0.1052), and wing discs (*Masc*: *p* = 0.0288, *BmIMP*^*M*^: *p* = 0.0057) (Fig 3B). The male-type *Bmdsx* (*Bmdsx*^*M*^) variants were also detected in all three tissues of *BmAgo3* KO female larvae (Fig 3C), indicating that BmAgo3 is involved in sex determination in both gonads and non-gonadal somatic tissues.

We next investigated the effects of *Siwi* KO on sex determination. As we failed to obtain fifth instar female larvae of *Siwi* KO (Fig 1D), we used total RNA prepared from the whole body of third instar larvae. *Masc* mRNA levels increased in *Siwi* KO female larvae, whereas its expression did not change in *BmAgo3* KO female larvae (Fig 3D). *BmIMP*^*M*^ mRNA levels slightly increased in *Siwi* KO female larvae, but not statistically significant (Fig 3D). Its expression did not change in *BmAgo3* KO female larvae. Consistently, *Bmdsx*^*M*^ expression was clearly detected in all *Siwi* KO female larvae, together with *Bmdsx*^*F*^ expression (Fig 3E), but only faintly in some *BmAgo3* KO larvae. These results indicate that Siwi loss induces masculinization in female larvae, which is more apparent compared to that induced by *BmAgo3* KO. The degree of derepression of *Masc* mRNA and subsequent masculinization by loss of BmAgo3 appears to vary in tissues and developmental stages (Fig 3B–E).

### Dysregulation of global gene expression by *BmAgo3* and *Siwi* KO

**Fig 4:**
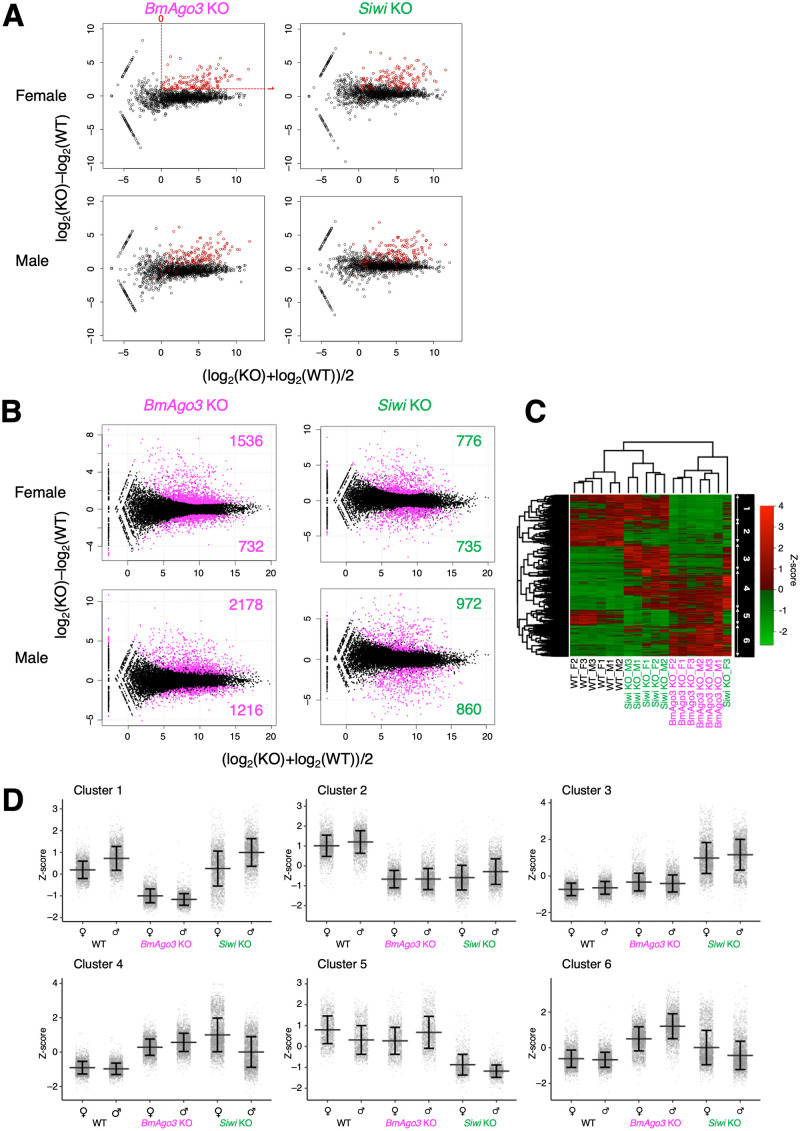
Dysregulation of global gene expression in the whole body in *BmAgo3* and *Siwi* KO. (A) Transposon derepression by *BmAgo3* and *Siwi* KO. RNA-seq libraries were prepared from total RNA extracted from the whole body of two third instar larvae. MAplots were created using the average of triplicate repeats compared to the WT. Each dot indicates a transposon; red dots indicate transposons at M (y-axis) > 1 and A (x-axis) > 0 in *BmAgo3* KO females. The axes show: A (x-axis) = (log_2_(TPM in KO) + log_2_(TPM in WT))/2. M (y-axis) = log_2_(TPM in KO)–log_2_(TPM in WT). Derepressed transposons in *BmAgo3* KO females were also depressed in *Siwi* KO females and in *Siwi* and *BmAgo3* KO males. (B) Differentially expressed genes (DEGs) in *BmAgo3* and *Siwi* KO larvae. MAplots were created using the average of triplicate repeats compared to the WT. Each dot indicates a gene; magenta dots indicate DEGs with variable expression at false discovery rate (FDR) of < 0.001. FDR was calculated using TCC R package [56]. (C) Expression patterns of genes showing statistically significant expression changes in any comparisons (magenta dots in (B)). TPMs of all DEGs in *BmAgo3* and *Siwi* KO larvae indicated by magenta dots in (B) were normalized by Z-score and clustered into six major clusters using Heatplus R package. (D) Plots of Z-scores of genes belonging to each of the six clusters. Three plots per gene are shown as they were analyzed in triplicate. The bar graph shows the average ± SD of all points.

To understand the mechanisms by which *BmAgo3* and *Siwi* KO result in unexpected abnormal phenotypes in somatic tissues in addition to the gonads, we performed RNA-seq analysis using whole body samples from WT, *BmAgo3* KO and *Siwi* KO third instar larvae. We first examined changes in transposon expression and found the same set of derepressed transposons in *BmAgo3* and *Siwi* KO males and females (Fig 4A). Next, we examined expression changes in all *B*. *mori* genes annotated as gene models and identified a huge amount of differentially expressed genes (DEGs) in *BmAgo3* and *Siwi* KO larvae (Fig 4B). The DEGs were clustered into six major groups (Fig 4C and 4D), e.g., transcripts commonly down-regulated or up-regulated upon *Siwi* or *BmAgo3* KO were defined as cluster 2 and 4, respectively. Gene ontology (GO) analysis revealed that genes in cluster 2 had GOs for “oxidation-reduction process” and “heme binding,” and most were *Cytochrome P450* (*CYP*) genes (S3 Table and S5 Fig). A transcriptional heat map of all *CYP* genes confirmed the groups containing down-regulated *CYP* genes in both *Siwi* and *BmAgo3* KO larvae (S6 Fig). Moreover, a phylogenetic tree of *CYP* genes revealed a downregulation tendency in genes belonging to specific branches (S7 Fig). Three genes annotated with β-glucosidase activity were included in the top 10 down-regulated genes (S4 Table). A heat map of the expression levels of all *B*. *mori β-glucosidase* genes showed numerous down-regulated *β-glucosidase* genes in *Siwi* and/or *BmAgo3* KO larvae (S8 and S9 Figs). On the other hand, many genes commonly up-regulated in *Siwi* and *BmAgo3* KO larvae (clusters 3 and 4) had GO terms for “DNA integration,” which results from a certain number of transposons contaminated in gene models (S3 Table and S5B Fig). This was confirmed by the fact that highly up-regulated genes included many transposons (S3 Table).

To examine the tissue-specific effects of *Siwi* and *BmAgo3* KO in somatic tissues, we identified genes specifically expressed in each tissue of fifth instar larvae. We found that 78% of ovary-specific genes were down-regulated (1 < log_2_(Fold Change)) in *BmAgo3* KO larvae (S10A Fig). These down-regulated genes were enriched in GO terms for “mitochondrion” (S5 Table). Fat body-specific genes were enriched in GOs with annotations such as “oxidation-reduction process” and “heme binding” (S5 Table and S11 Fig). This was similar to cluster 2, which contained down-regulated genes in *Siwi* and *BmAgo3* KO third instar larvae (S3 Table). Violin plots showed down-regulated fat body-specific genes in both *Siwi* and *BmAgo3* KO third instar larvae, which was not observed in all genes, or in ovary- and wing disc-specific genes (S10B Fig). We also found that down-regulated wing disc-specific genes had GO terms for chitin production (S5 Table and S11 Fig), suggesting that the defect in wing development might be caused by chitin production failure.

## Discussion

The piRNA pathways have been extensively studied in *Drosophila*'s *piwi*, *aub*, and *ago3* mutants which can develop to adult flies but they are sterile or semi-sterile [29,33–35]. In this study, we established *Siwi* (*aub* functional orthologue) and *BmAgo3* (*ago3* functional orthologue) KO mutants in *B*. *mori* and found that both *Siwi* and *BmAgo3* KO larvae do not develop to adult moths, demonstrating that unlike *Drosophila*, *B*. *mori* piRNA plays a role in the normal development of non-gonadal somatic tissues as well as gonadal tissues. *Siwi* and *BmAgo3* KO larvae grew slowly and exhibited defects in wing, ovary, and testis development. These developmental defects, with the exception of oogenesis arrest, were not observed in previous *Siwi* and *BmAgo3* KO studies using the somatic transgenic CRISPR/Cas9 method [46,50]. *Siwi* and *BmAgo3* germline KO mutants generated in this study clearly demonstrated the role of piRNAs in the development of gonads and non-gonadal somatic tissues in *B*. *mori*.

As described in the Introduction, it is suggested that somatic piRNAs targeting transposons and mRNAs are common among arthropods [42]. As the ping-pong cycle is not active in *Drosophila* non-gonadal somatic tissues, functional examination of *Aub* and *Ago3* orthologues in non-*Drosophila* insects has been required to discover other unknown functions of somatic piRNAs in insects. Using loss-of-function mutants of two PIWI genes, this study showed that somatic piRNAs are generated via the ping-pong cycle and probably ensure genome integrity against transposon mobilization like in gonadal tissues of *B*. *mori*. Although *B*. *mori* somatic piRNAs originate from a lower number of transposons compared to those in ovarian piRNAs, the piRNA-producing loci are not clearly separated into somatic and germline ones, in contrast with the *Drosophila* system [57].

In *Drosophila*, *piwi* mutants showed piRNA depletion in the fat body, resulting in increased transposon expression and DNA damage [36]. The mutants also exhibited reduced accumulation of lipid droplets in the fat body. Accordingly, it appears that these defects cause loss of fat body function and a short-lived phenotype. Unlike *Drosophila* Piwi, which localizes in the nucleus, *B*. *mori* Siwi (*B*. *mori* functional Aub orthologue) and BmAgo3 (*B*. *mori* functional Ago3 orthologue) are found in cytoplasmic RNP granules “nuage” [58,59]. Although Siwi and BmAgo3 are not involved in chromatin regulation in the nucleus, some *Siwi* and *BmAgo3* KO phenotypes are relatively similar to those of *Drosophila piwi* mutants. In *Drosophila*, Aub and Ago3 are crucial for repressing transposon activity by cleaving transposon RNA in the cytoplasm of germ cells, whereas they are not likely involved in transposon repression in somatic cells. In *Drosophila* somatic cells, Piwi plays a role in repressing transposon activity in the nucleus, but its role is limited [36,60–62]. A recent study revealed that the piRNA and siRNA pathways appear to play a minor role in somatic cells to repress transposons, and that zinc-finger transcriptional regulator Histone Nuclear Factor P (Hinfp) is critical for silencing of most transposons in somatic tissues by maintaining Histone1 expression [63]. Interestingly, Siwi and BmAgo3 can post-transcriptionally repress the majority of transposons in non-gonadal somatic tissues (Figs 2F, 4A and S4 Table), suggesting that piRNA pathways are important for repressing transposon activity in non-gonadal somatic tissues in *B*. *mori*.

Although insects have variable numbers of *Aub*/*Piwi-like* genes in their genome, two *Aub*/*Piwi-like* genes were detected in a large proportion (approximately 40%) of the 174 arthropods [38]. Duplication of *Aub*/*Piwi-like* gene has occurred in *L*. *migratoria* (*Piwi1* and *Piwi2*) [38], the pea aphid, *Acyrthosiphon pisum* (*Piwi1-8*) [64][65], the yellow fever mosquito, *Aedes aegypti* (*PIWI1-9*) [66], and flies (*piwi* and *Aub*) [31]. Lewis *et al* estimated that divergence of Aub and Piwi subclades has occurred at the base of the Brachycera between 182 and 156 mega-annum (Ma) [31]. On the other hand, most insect species have a single copy of *Ago3* gene [42]. Recent progress in next-generation sequencing has confirmed the existence of piRNAs along with the expression of *Aub*/*Piwi-like* and *Ago3* genes in arthropod somatic tissues, suggesting the importance of the piRNA pathways in non-gonadal somatic tissues [42]. *B*. *mori* has a single copy of both *Aub*/*Piwi-like* gene (*Siwi*) and *Ago3* gene (*BmAgo3*). In addition, piRNA biogenesis in *B*. *mori* has been well characterized using the ovary-derived cell line BmN-4. Therefore, *B*. *mori* is one of the useful materials for studying piRNA biogenesis and functions in somatic tissues. Using *Siwi* and *BmAgo3* germline mutants, we experimentally demonstrated that the ping-pong cycle plays a crucial role in transposon silencing and normal gene expression in non-gonadal somatic tissues. Our results suggest that in many insects, somatic piRNAs contribute to transposon repression and normal gene expression. In fact, at least 2% of somatic piRNAs were mapped to transposons in most arthropod species examined [42]. Furthermore, piRNA-mediated sex determination systems evolved in some lepidopteran insects may provide insights into the evolution of the somatic piRNA pathway from global transposon silencing to the regulation of a specific physiological process [51,67].

It is well known that disruption of piRNA pathways results in developmental defects in gonadal tissues, where piRNAs are abundantly accumulated. Transposon derepression is commonly observed in gonadal tissues, but the molecular mechanisms of developmental defects have remained unclear. In this study, we showed that piRNAs greatly contribute to transposon repression and normal development of both gonadal tissues (ovary and testis) and non-gonadal somatic tissues (fat body and wing disc) in *B*. *mori*. These results indicate a common, but until now unknown link between piRNAs and tissue homeostasis in gonadal tissues and non-gonadal somatic tissues. *B*. *mori* is a suitable model to explore this missing link by enabling the use of multiple piRNA-producing tissues and piRNA pathway mutants.

*Siwi* KO resulted in more severe phenotypes compared to *BmAgo3* KO. Two possible mechanisms may explain the different phenotypes observed in these mutants. First, Siwi–piRNA complexes may be more important than BmAgo3-piRNA complexes for suppressing transposon activity and/or regulating protein-coding genes in non-gonadal somatic tissues. In fact, transposons were more derepressed and protein-coding gene expression fluctuated more in *Siwi* KO larvae (Fig 4A, 4B, 4D and S4 Table). A second hypothesis is that Siwi has functions other than piRNA-mediated target silencing. *Drosophila* Piwi is involved in stem cell maintenance through piRNA-independent mechanisms [68]. In addition, human PIWIL1’s role in some cancers is reported to be independent of gene silencing [69,70]. Future studies need to address Siwi’s role in the development of somatic tissues in *B*. *mori*.

piRNA-mediated gene silencing occurs in non-gonadal somatic tissues as well as gonadal tissues (S2 Table). In addition, transcriptome analysis identified a huge amount of up-regulated and down-regulated genes in *BmAgo3* and *Siwi* KO larvae. Among them, commonly up-regulated and down-regulated genes in *Siwi* and *BmAgo3* KO larvae were classified. Lower transcripts of *CYP* and *β-glucosidase* family genes were prominent in both KO larvae. Insect CYPs play an important role in physiological functions such as hormone biosynthesis and detoxification of plant allelochemicals and insecticides, at all life stages [71,72]. Reduced expression of *CYP* family genes possibly causes developmental defects and weakness in both *Siwi* and *BmAgo3* KO larvae. Kang *et al* [73] reported that, in a lethal silkworm mutant in the fourth instar (*l-4i*), the reduced mRNA level of *KWMTBOMO10213*, a *β-glucosidase* gene, causes lethality at the fourth instar larvae due to energy depletion, supporting the idea that lower transcription of *β-glucosidase* family genes leads to developmental delay in both *Siwi* and *BmAgo3* KO larvae. Moreover, reduced mRNA levels of genes required for chitin production (cuticular protein genes) in the wing disc possibly explain failed wing development, because wing disc development is heavily dependent on chitin biosynthesis [74]. Why does BmAgo3 or Siwi loss lead to decreased expression of genes that belong to the same family? *CYP*, *β-glucosidase*, and cuticular protein genes are highly divergent due to gene duplication in insects (S7 and S9 Figs). Therefore, a common transcriptional regulation pathway(s) shared by these gene families may be affected by the failure of somatic piRNA biogenesis.

The most striking feature of *B*. *mori* piRNA is that a single piRNA determines femaleness [51]. The *Fem* piRNA–Siwi complex cleaves Z chromosome-derived *Masc* mRNA, while the *Masc* piRNA–BmAgo3 complex cleaves *Fem*, leading to *Fem* piRNA production. In the previous study, *Fem* piRNA and *Masc* piRNA pairs were detected in early embryos, ovaries, and ovary-derived cultured cells. In this study, we found that this *trans* ping-pong system is active in fat body and wing disc tissues as well as the ovary. We also found that *Fem* and *Masc* piRNAs are almost absent in *BmAgo3* KO tissues, suggesting that most of the two piRNAs are amplified via the ping-pong cycle. In addition, *Siwi* and *BmAgo3* KO larvae lost normal Siwi and BmAgo3 proteins (Figs 1C and S1). However, masculinization, i.e., inhibition of feminization, did not occur completely in *Siwi* and *BmAgo3* KO females (Fig 3B–E). This may be due to the regulation system of the *Masc* gene. The gene dosage of Z-linked *Masc* is different between the sexes, resulting in sexual difference in the *Masc* mRNA level. *Fem* piRNA is utilized to fine-tune *Masc* expression to inhibit the Masc-mediated cascade completely. Such a piRNA-mediated *Masc* expression has been shown to evolve independently in a lepidopteran insect other than *B*. *mori* [67]. Since *Fem* is the multicopy precursor of *Fem* piRNA on the W chromosome, it is technically difficult to generate a complete *Fem* KO strain. With successful complete KO generation, we could understand the role of *Fem* on sexual differentiation and somatic cell fate in *B*. *mori*.

## Materials and methods

### Silkworm strains

In this study, we used the non-diapause strain N4 maintained in our laboratory. All larvae were fed with fresh mulberry leaves or artificial diet SilkMate PS (NOSAN) under a continuous 12-h light/darkness cycle at 25°C. Mutant strains were maintained by crossing between heterozygous mutant moths (−/+) as no homozygous mutant moths (−/−) were obtained. Heterozygous mutant moths (−/+) were identified by T7ENI cleavage assay or heteroduplex mobility assay as described below. Phenotypes were photographed with an Olympus DP70 camera under an Olympus SZX12 microscope.

### CRISPR/Cas9-mediated mutagenesis

We prepared sgRNAs according to a previously reported method [75]. Specific sgRNA target sequences were searched using ZiFiT Targeter [76]. The primers used for sgRNA transcription *in vitro* are listed in S6 Table. A mixture of sgRNA (400 ng/μL) and Cas9 protein (600 ng/μL; NIPPON GENE) in injection buffer (100 mM KOAc, 2 mM Mg(OAc)_2_, 30 mM HEPES-KOH; pH 7.4) was injected into each egg within 2–4 h after oviposition [77]. The injected embryos were incubated at 25°C in a humidified Petri dish until hatching. Fertile injected individuals were crossed with non-injected individuals to obtain G_1_ broods. We screened them by T7 endonuclease I (T7ENI) assay or heteroduplex mobility assay as described below, and identified G_1_ broods with mutant alleles transmitted from G_0_. We maintained the screened broods and established the mutant line according to the mating scheme reported by Daimon *et al* [78].

### T7ENI cleavage assay and heteroduplex mobility assay

To detect mutations at the target site, genomic DNA was extracted from G_1_ neonate larvae or a leg of an adult moth using the hot sodium hydroxide and tris (HotSHOT) method [79]. Genomic DNA was also prepared from a third instar larva used in total RNA extraction described below using TRIzol reagent (Invitrogen), according with the manufacturer’s protocol [80]. Genomic PCR was conducted using KOD One (TOYOBO) with the primer sets listed in S6 Table under the following conditions: 40 cycles of denaturation at 98°C for 10 s, annealing at 60°C for 5 s, and extension at 68°C for 5 s. The PCR product was annealed under the following conditions: 95°C for 10 min, 85°C for 1 min, 75°C for 1 min, 65°C for 1 min, 55°C for 1 min, 45°C for 1 min, 35°C for 1 min, and 25°C for 1 min, followed by incubation at 4°C. The annealed PCR products were cleaved by T7ENI (NEB) at 37°C for 1 h. The cleavage products were detected by agarose gel electrophoresis. For the heteroduplex mobility assay, the annealed PCR products were electrophoresed using the MultiNA microchip electrophoresis system (SHIMADZU) with the DNA-500 reagent kit [81,82].

### DNA sequencing

To identify mutations at the target site, the above PCR products were sequenced with a BigDye Terminator v3.1 Cycle Sequencing Kit (Applied Biosystems) and ABI PRISM 3130*xl* Genetic Analyzer (Applied Biosystems). The primers are listed in S6 Table.

### Reverse transcription (RT)-PCR and RT-quantitative PCR (RT-qPCR)

Total RNA was prepared from the whole body of a third instar larva or tissues from fifth instar larvae using TRIzol reagent according with the manufacturer’s protocol and subjected to reverse transcription using avian myeloblastosis virus reverse transcriptase with an oligo-dT primer (TaKaRa). RT-PCR was conducted using KOD FX Neo under the following conditions: 94°C for 2 min; 40 cycles of 98°C for 15 s, 60°C for 30 s, and 68°C for 30 s; followed by 68°C for 2 min. *Bmdsx* splicing patterns were examined by RT-PCR with the primers listed in S6 Table. RT-qPCR was performed using KAPA SYBR FAST qPCR kit (KAPA Biosystems) and StepOnePlus Real-Time PCR System (Applied Biosystems). Molecular sexing was performed by RT-qPCR analysis using a W-specific marker, *Fem* [51]. The primers are listed in S6 Table.

### Western blot analysis

A whole cell extract obtained from a third instar larva was used for SDS-PAGE, followed by western blotting. The Anti-Siwi and anti-BmAgo3 polyclonal antibodies were described previously [12]. Anti-α-Tubulin monoclonal antibody (clone B-5-1-2) was purchased from Sigma-Aldrich. Chemiluminescence was induced by a Luminata Forte Western HRP Substrate (Millipore) and images were acquired by an Amersham Imager 600 (GE Healthcare).

### Small RNA cloning

Small RNA libraries were prepared from 20–50 nt sequences according to Zamore lab’s open protocol (https://www.dropbox.com/s/r5d7aj3hhyaborq/) [83] with some modifications. The 3′ adapter was conjugated with an amino CA linker instead of dCC at the 3′ end (GeneDesign) and adenylated using a 5′ DNA adenylation kit at the 5′ end (NEB). To reduce ligation bias, four random nucleotides were included in the 3′ and 5′ adapters [(5′-rAppNNNNTGGAATTCTCGGGTGCCAAGG/amino CA linker-3′) and (5′-GUUCAGAGUUCUACAGUCCGACGAUCNNNN-3′)] and adapter ligation was performed in the presence of 20% PEG-8000. After 3′ adapter ligation at 16°C for ≥16 h, RNAs were size-selected by urea PAGE. For RNA extraction from the polyacrylamide gel, a ZR small RNA PAGE Recovery Kit (ZYMO Research) was used. Small RNA libraries were sequenced using the Illumina HiSeq 4000 platform to obtain 50 nt single-end reads.

### Sequence analysis of cloned small RNAs

The 3′-adapter sequences were identified and removed, allowing for < 2 mismatches. Reads < 20 nt or > 40 nt were excluded, thereby obtaining reads between 20–40 nt. The mapping of small RNAs to *B*. *mori* transposons [84] and *B*. *mori* gene models [85] was conducted using bowtie [86]. For each library, normalization was performed using total transposon mapped reads (without rRNA-derived repeats) and again using the 10 most abundant miRNA reads [14,87]. SAM files were converted to BAM files using SAMtools [88], then to BED files; each nucleotide’s coverage was calculated using BEDTools [89].

### RNA-seq analysis

For RNA prepared from fifth instar larval tissues, strand-specific RNA-seq libraries were prepared using SureSelect Strand-Specific RNA Reagent Kit (Agilent Technologies) and sequenced using the Illumina HiSeq 4000 platform. Meanwhile, for RNA prepared from a third instar larval whole body, strand-specific RNA-seq libraries were prepared using NEBNext Ultra Directional RNA Library Prep Kit for Illumina (New England BioLabs) and sequenced using the Illumina NovaSeq 6000 platform by Novogene. Poly-A selection was carried out in both libraries’ construction. Mapping strand-specific RNA reads to *B*. *mori* gene models [85] and transposons [84] was conducted using HISAT2 [90]. Reads that could be aligned to gene models up to one mismatch were used for normalization. SAM files were converted to BAM files using SAMtools [88], then to BED files; the coverage of each nucleotide was calculated using BEDTools [89].

## Supporting information

S1 Table All delayed larvae possessed homozygous KO alleles as shown by genotyping. (XLSX)

S2 Table Up-regulated genes in *BmAgo3* KO tissues. (XLSX)

S3 Table GO analysis of differentially expressed genes in *BmAgo3* and *Siwi* KO mutants (Fig 4C cluster). (XLSX)

S4 Table Top 10 genes with altered expression in *BmAgo3* and *Siwi* KO. (XLSX)

S5 Table GOs enriched in tissue-specific genes and corresponding expression changes. (XLSX)

S6 Table List of primers. (XLSX)

S1 Fig CRISPR/Cas9-mediated *BmAgo3* and *Siwi* knockout. (A) *BmAgo3* sequences surrounding the sgRNA target site and predicted protein structures. The target sgRNA site is underlined, and the proto-spacer adjacent motif (PAM) highlighted by a gray box. The cleavage site is shown by an arrowhead. The deleted (−) and inserted (small letters) sequences are shown near the cleavage site. The rounded rectangles indicate the location of protein domains corresponding to Siwi proteins [91]. (B) *Siwi* sequences surrounding the sgRNA target site and predicted protein structures. The target sgRNA site is underlined, and the PAM highlighted by a gray box. The cleavage site is shown by an arrowhead. The deleted (−) sequences are shown near the cleavage site. The rounded rectangles indicate the location of protein domains. The PAZ domain in the *Siwi* KO mutant is slightly truncated (gray rounded rectangles).
(TIF)

S2 Fig Developmental delay in *BmAgo3* and *Siwi* KO mutant larvae. (A) The third instar larvae of *BmAgo3* KO mutants. (B) The third instar larvae of *Siwi* KO mutants. About a quarter of larvae exhibited developmental delay at the larval stage. Scale bars, 1 cm.
(TIF)

S3 Fig Detailed phenotypes of *BmAgo3* and *Siwi* KO mutant pupae. (A) Pupal weight of *BmAgo3* and *Siwi* KO mutants. No female pupae were obtained from the *Siwi* KO mutant. Bars indicate means ± SE. The number indicates the sample size. Asterisks indicate statistical significance in Mann-Whitney test (*p* < 0.05). (B) Enlarged pictures of head and leg morphologies in *BmAgo3* and *Siwi* KO mutant pupae. Scale bars, 2 mm.
(TIF)

S4 Fig piRNAs specifically expressed in the fat bodies derived from the *SP1* gene. (A) 5’-end positions of piRNAs mapped onto *SP1* (*KWMTBOMO13992*) and the precursor of *SP1* partner piRNA (*KWMTBOMO03023*, putative transposon (TE)) using piRNA tissue libraries. The positive and negative directions of the y-axis indicate piRNAs mapped onto the sense and antisense strands, respectively. Black: WT, green: *BmAgo3* KO. (B) Positional relationship between *SP1* piRNA and partner piRNA that can form the 5’-end of *SP1* piRNA (top). The *SP1* partner piRNA has a reverse complementary sequence to the *KWMTBOMO13992* (*SP1*) mRNA from the 2^nd^ to the 18^th^ base, and can induce *SP1* mRNA cleavage. Relationship between *SP1* piRNA and the precursor of *SP1* partner piRNA (*KWMTBOMO03023*, putative transposon (TE)) (bottom). *SP1* piRNA is not complementary to *KWMTBOMO03023*. (C and D) Expression levels of *SP1* piRNA (C) and *SP1* partner piRNA (D) in each small RNA library. (E and F) Expression levels of *SP1* (E) and the precursor of *SP1* partner piRNA (F) in each mRNA library.
(TIF)

S5 Fig GO analysis of differentially expressed genes in *BmAgo3* and *Siwi* KO third instar larvae. (A and B) GO analysis for molecular function (A) and biological process (B). Bonferroni-adjusted *p* values (–log_10_) and selected gene numbers in each GO shown by gray bars and yellow circles, respectively.
(TIF)

S6 Fig *CYP* genes commonly down-regulated in *BmAgo3* and *Siwi* KO. Clustering of expression variation patterns of *B*. *mori CYP* genes in mRNA libraries in the whole body of third instar larvae. TPMs of *B*. *mori CYP* genes were normalized by Z-score and clustered using Heatplus R package. F: female, M: Male.
(TIF)

S7 Fig Phylogenetic tree of *B*. *mori CYP* genes. Phylogenetic tree using amino acid sequences of *B*. *mori* Cytochrome P450. Genes in red indicate *CYP* genes commonly down-regulated in the absence of PIWI protein. Gene models annotated as Cytochrome P450 were aligned using the MUSCLE program [92], and a phylogenetic tree was built using MEGA X software with the maximum likelihood method [93].
(TIF)

S8 Fig *β-glucosidase* genes commonly down-regulated in *BmAgo3* and *Siwi* KO. (A) Clustering of *B*. *mori β-glucosidase* gene expression patterns. Numerous *β-glucosidase* genes were commonly down-regulated in *BmAgo3* and *Siwi* KO. TPMs of *B*. *mori β-glucosidase* genes were normalized by Z-score and clustered using Heatplus R package. F: female, M: Male. (B) Alignment of amino acid sequences of *B*. *mori* β-glucosidases.
(TIF)

S9 Fig Phylogenetic tree of *β-glucosidase* genes. Phylogenetic tree using amino acid sequences of *B*. *mori* β-glucosidases. Black: *Bombyx mori*, red: *Drosophila melanogaster*, blue: *Tribolium castaneum*, yellow: *Danaus plexippus*. Gene models annotated as β-glucosidase were aligned using the MUSCLE program [92], and a phylogenetic tree was built using MEGA X software with the maximum likelihood method [93]. Numbers indicate bootstrap probabilities (%).
(TIF)

S10 Fig Expression changes of tissue-specific genes in *BmAgo3* and *Siwi* KO larvae. (A) Genes specifically expressed in the ovaries (OV), fat body (FB), and wing discs (WD) in fifth instar larvae were investigated for increased or decreased (1 < log_2_(Fold Change)) expression in *BmAgo3* KO. Each tissue-specific gene was defined as that whose TPM in the WT library of one tissue was more than twice as large as that in the libraries of the other two tissues. (B) Violin plots of tissue-specific gene expression patterns in the whole body of third instar larvae.
(TIF)

S11 Fig GO analysis of tissue-specific genes in *BmAgo3* KO larvae. (A and B) GO analysis for molecular function (A) and biological process (B) in the fat body (FB) and wing discs (WD). Bonferroni-adjusted *p* values (–log_10_) and selected gene numbers in each GO are shown by gray bars and yellow circles, respectively.
(TIF)

S1 Data Data underlying the reported results. (XLSX)

S1 Metadata Metadata underlying the reported results. (XLSX)
