# Supplementary figures and images for: Non-gonadal somatic piRNA pathways ensure sexual differentiation, larval growth, and wing development in silkworms
Source: PLoS Genet. 2023 Sep 21;19(9):e1010912. doi: 10.1371/journal.pgen.1010912 (PMC10513339; doi:10.1371/journal.pgen.1010912)

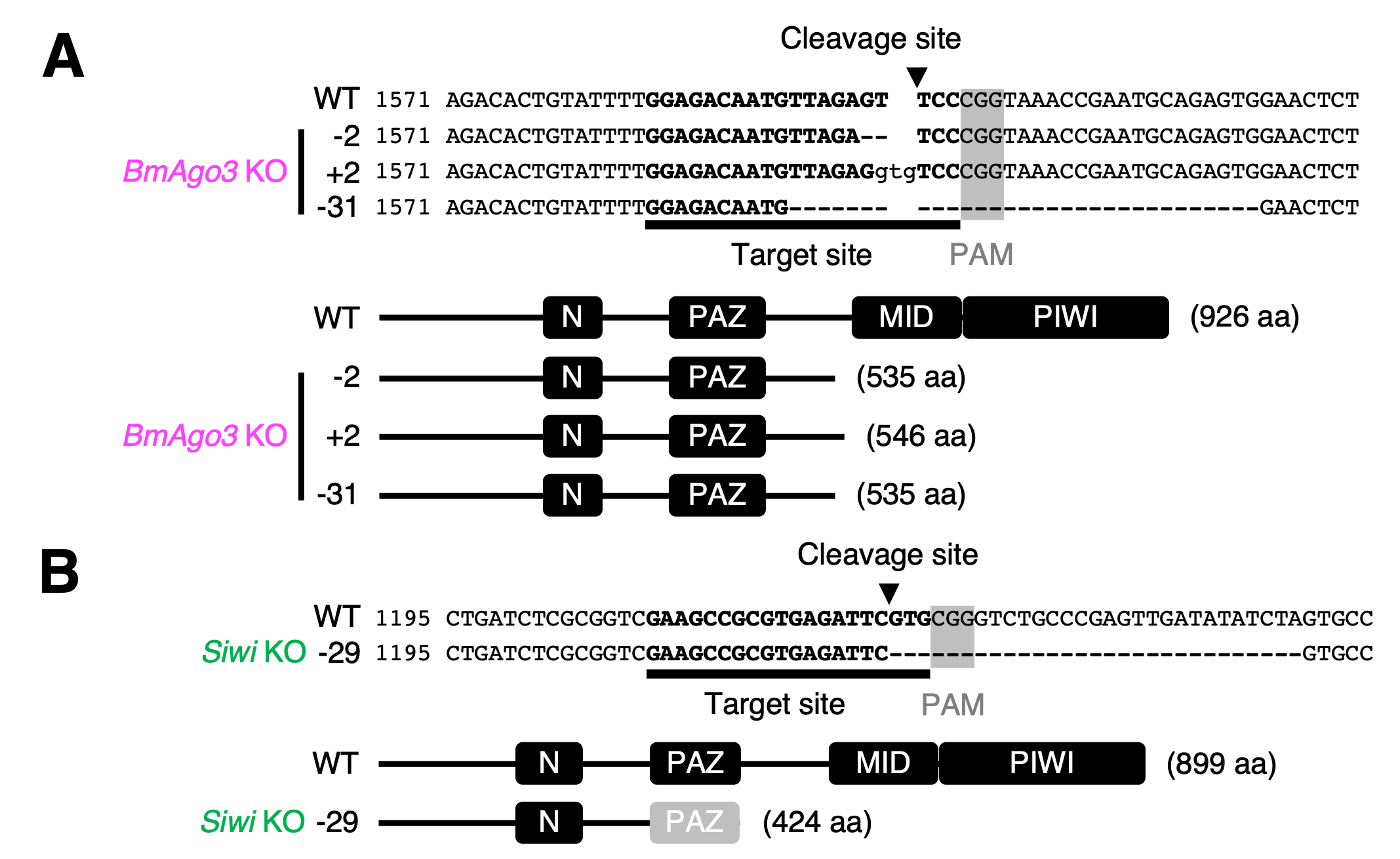

Supplement: S1 Fig — (A) BmAgo3 sequences surrounding the sgRNA target site and predicted protein structures. The target sgRNA site is underlined, and the proto-spacer adjacent motif (PAM) highlighted by a gray box. The cleavage site is shown by an arrowhead. The deleted (−) and inserted (small letters) sequences are shown near the cleavage site. The rounded rectangles indicate the location of protein domains corresponding to Siwi proteins [91]. (B) Siwi sequences surrounding the sgRNA target site and predicted protein structures. The target sgRNA site is underlined, and the PAM highlighted by a gray box. The cleavage site is shown by an arrowhead. The deleted (−) sequences are shown near the cleavage site. The rounded rectangles indicate the location of protein domains. The PAZ domain in the Siwi KO mutant is slightly truncated (gray rounded rectangles). (TIF) [file pgen.1010912.s007.tif]

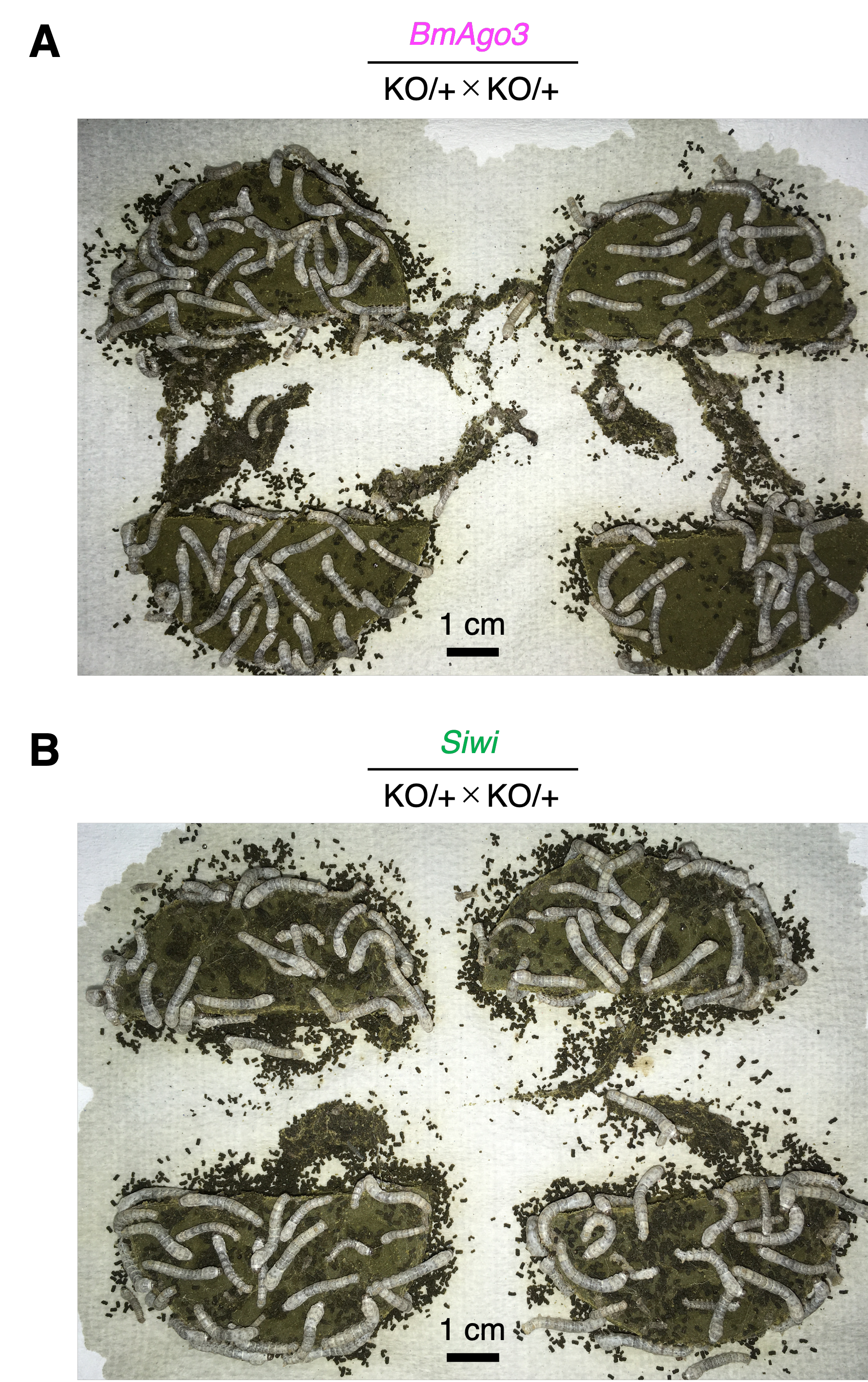

Supplement: S2 Fig — (A) The third instar larvae of BmAgo3 KO mutants. (B) The third instar larvae of Siwi KO mutants. About a quarter of larvae exhibited developmental delay at the larval stage. Scale bars, 1 cm. (TIF) [file pgen.1010912.s008.tif]

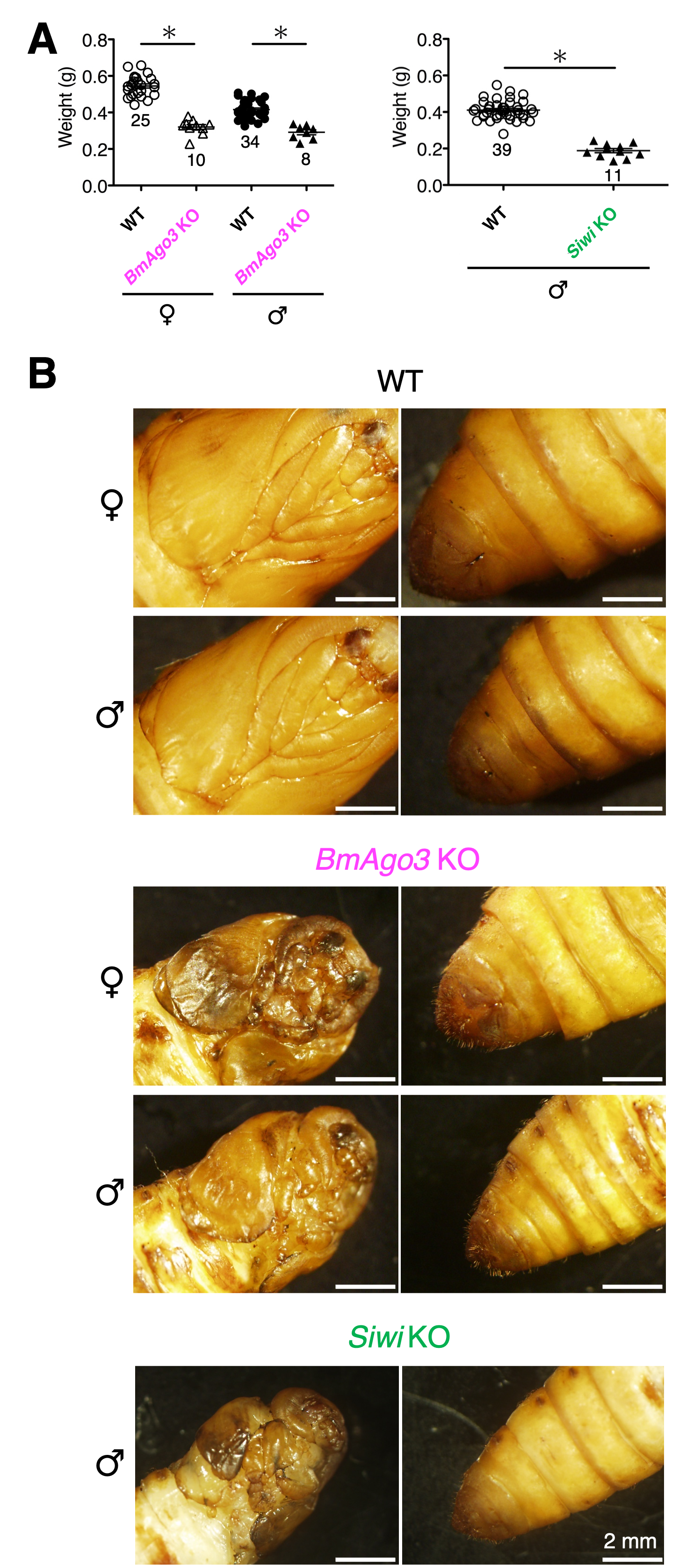

Supplement: S3 Fig — (A) Pupal weight of BmAgo3 and Siwi KO mutants. No female pupae were obtained from the Siwi KO mutant. Bars indicate means ± SE. The number indicates the sample size. Asterisks indicate statistical significance in Mann-Whitney test (p < 0.05). (B) Enlarged pictures of head and leg morphologies in BmAgo3 and Siwi KO mutant pupae. Scale bars, 2 mm. (TIF) [file pgen.1010912.s009.tif]

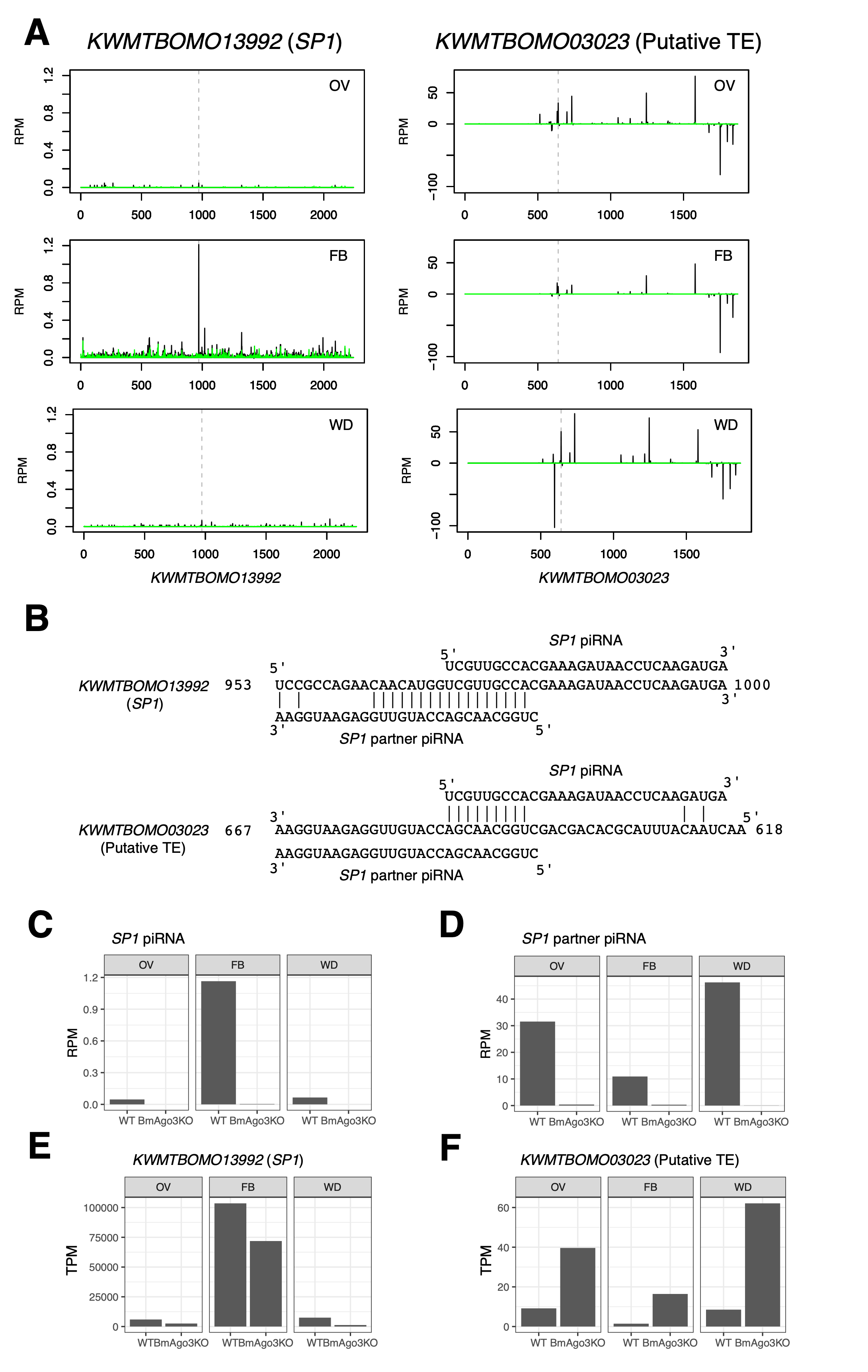

Supplement: S4 Fig — (A) 5’-end positions of piRNAs mapped onto SP1 (KWMTBOMO13992) and the precursor of SP1 partner piRNA (KWMTBOMO03023, putative transposon (TE)) using piRNA tissue libraries. The positive and negative directions of the y-axis indicate piRNAs mapped onto the sense and antisense strands, respectively. Black: WT, green: BmAgo3 KO. (B) Positional relationship between SP1 piRNA and partner piRNA that can form the 5’-end of SP1 piRNA (top). The SP1 partner piRNA has a reverse complementary sequence to the KWMTBOMO13992 (SP1) mRNA from the 2nd to the 18th base, and can induce SP1 mRNA cleavage. Relationship between SP1 piRNA and the precursor of SP1 partner piRNA (KWMTBOMO03023, putative transposon (TE)) (bottom). SP1 piRNA is not complementary to KWMTBOMO03023. (C and D) Expression levels of SP1 piRNA (C) and SP1 partner piRNA (D) in each small RNA library. (E and F) Expression levels of SP1 (E) and the precursor of SP1 partner piRNA (F) in each mRNA library. (TIF) [file pgen.1010912.s010.tif]

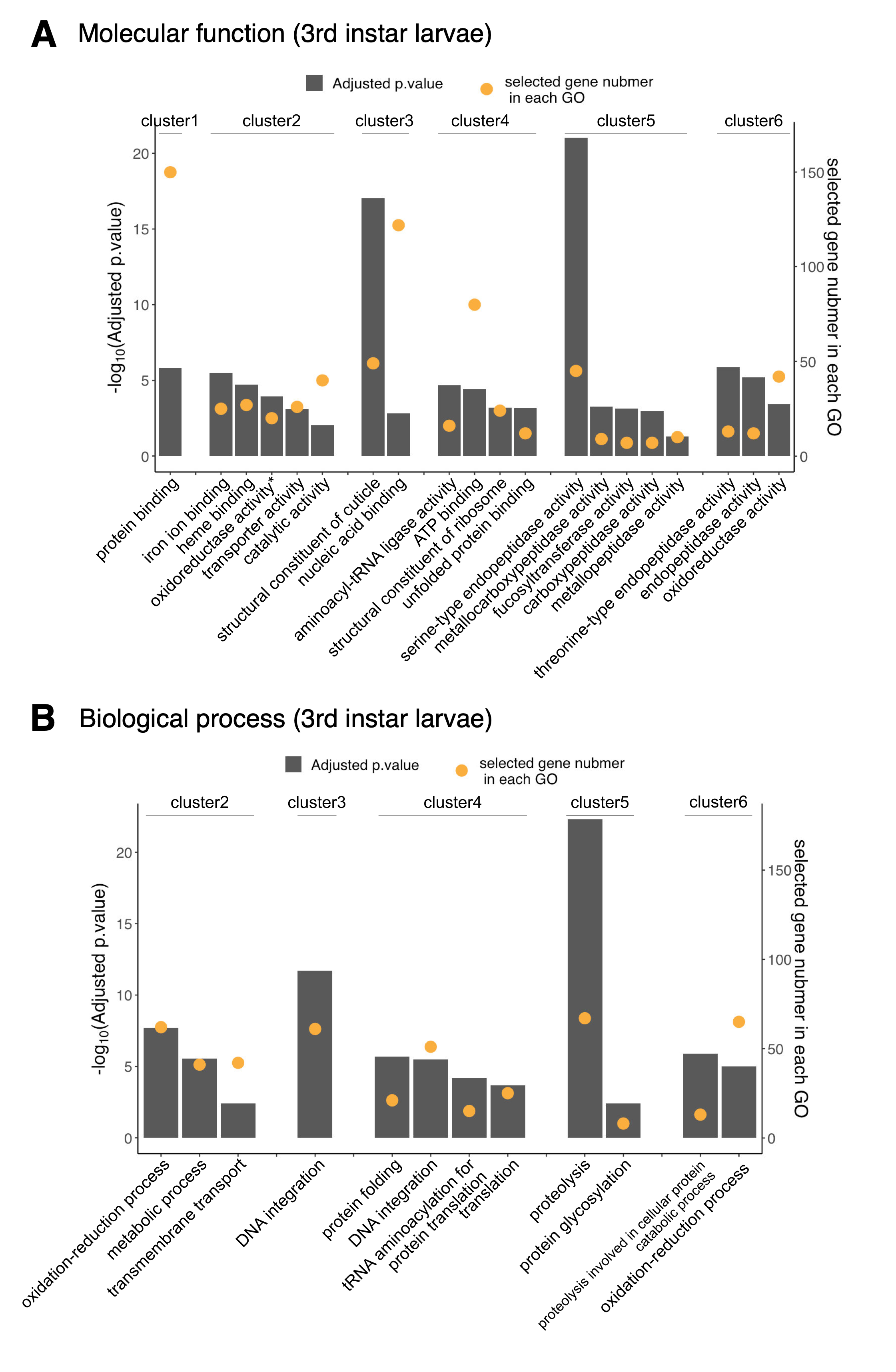

Supplement: S5 Fig — (A and B) GO analysis for molecular function (A) and biological process (B). Bonferroni-adjusted p values (–log10) and selected gene numbers in each GO shown by gray bars and yellow circles, respectively. (TIF) [file pgen.1010912.s011.tif]

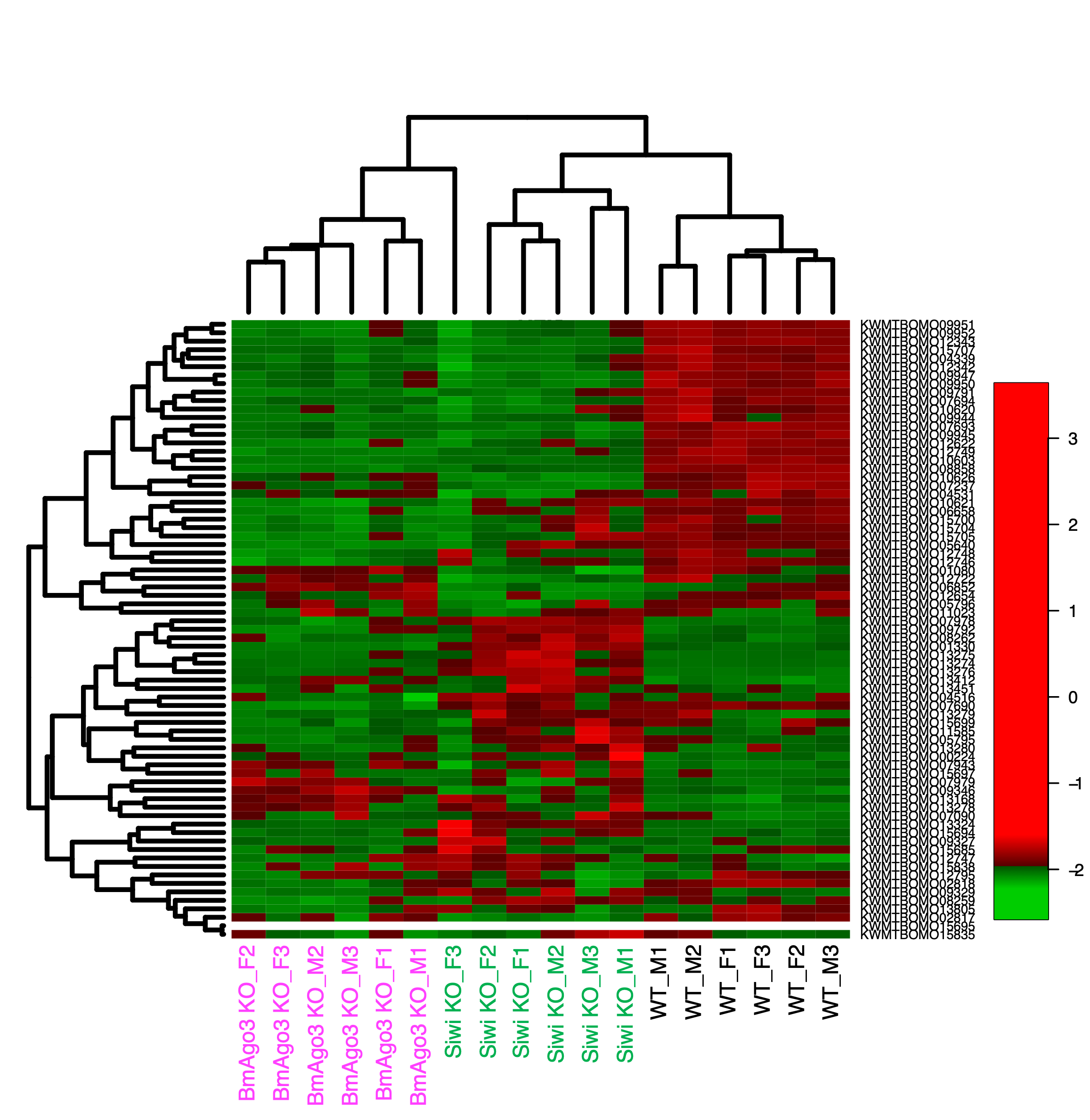

Supplement: S6 Fig — Clustering of expression variation patterns of B. mori CYP genes in mRNA libraries in the whole body of third instar larvae. TPMs of B. mori CYP genes were normalized by Z-score and clustered using Heatplus R package. F: female, M: Male. (TIF) [file pgen.1010912.s012.tif]

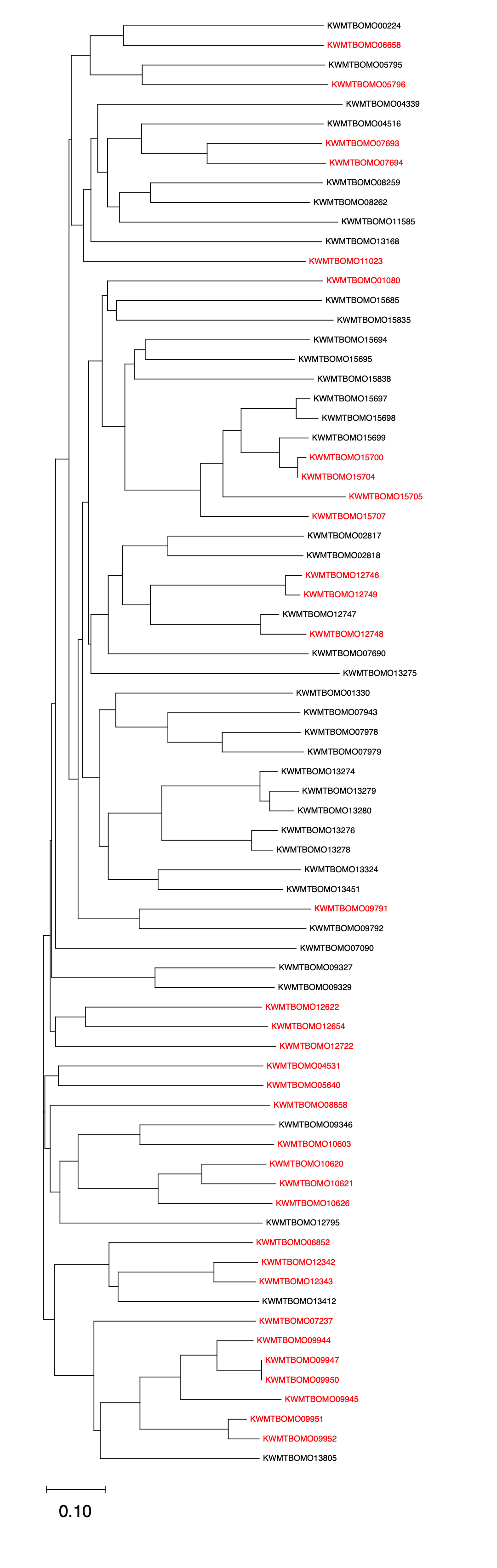

Supplement: S7 Fig — Phylogenetic tree using amino acid sequences of B. mori Cytochrome P450. Genes in red indicate CYP genes commonly down-regulated in the absence of PIWI protein. Gene models annotated as Cytochrome P450 were aligned using the MUSCLE program [92], and a phylogenetic tree was built using MEGA X software with the maximum likelihood method [93]. (TIF) [file pgen.1010912.s013.tif]

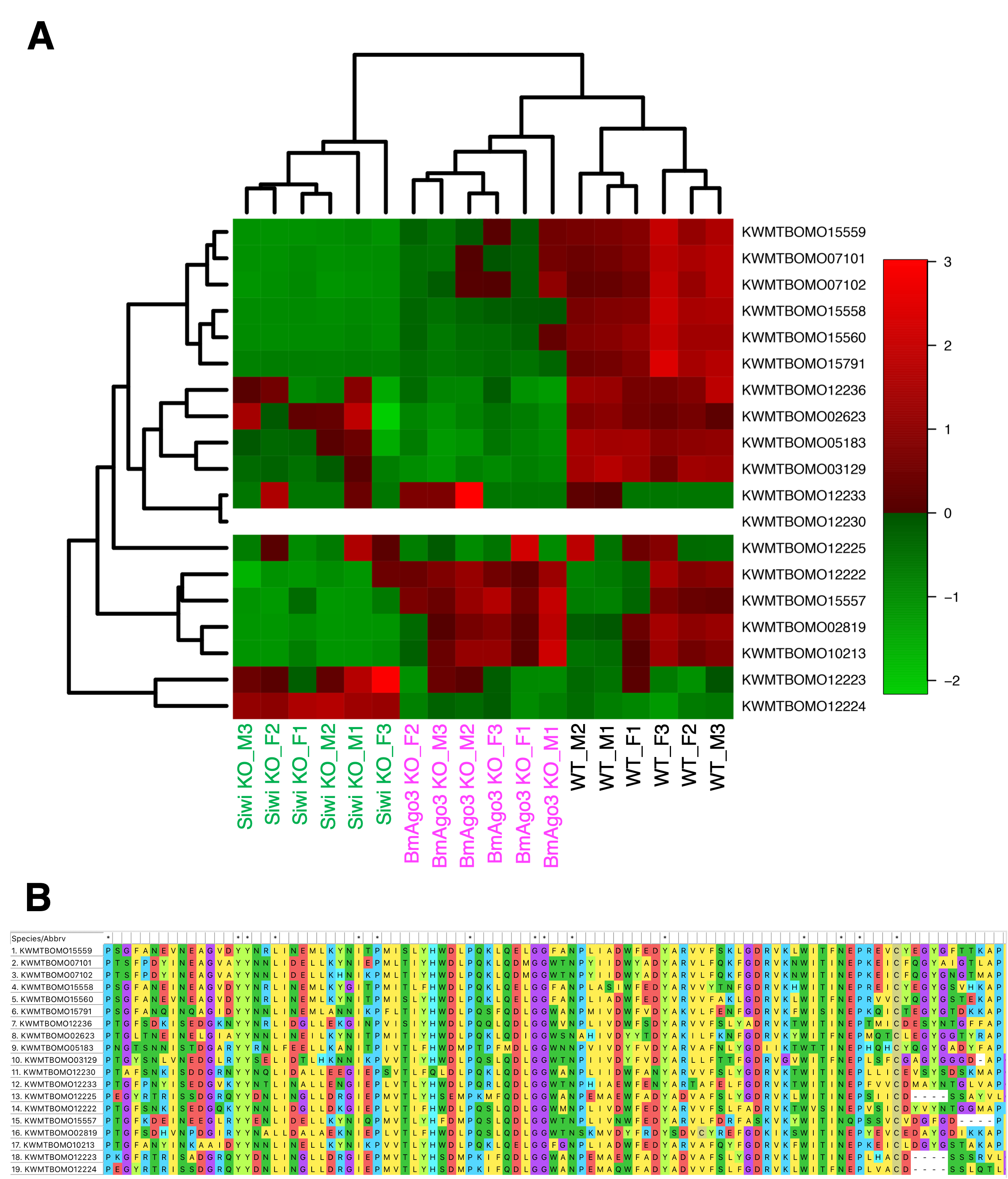

Supplement: S8 Fig — (A) Clustering of B. mori β-glucosidase gene expression patterns. Numerous β-glucosidase genes were commonly down-regulated in BmAgo3 and Siwi KO. TPMs of B. mori β-glucosidase genes were normalized by Z-score and clustered using Heatplus R package. F: female, M: Male. (B) Alignment of amino acid sequences of B. mori β-glucosidases. (TIF) [file pgen.1010912.s014.tif]

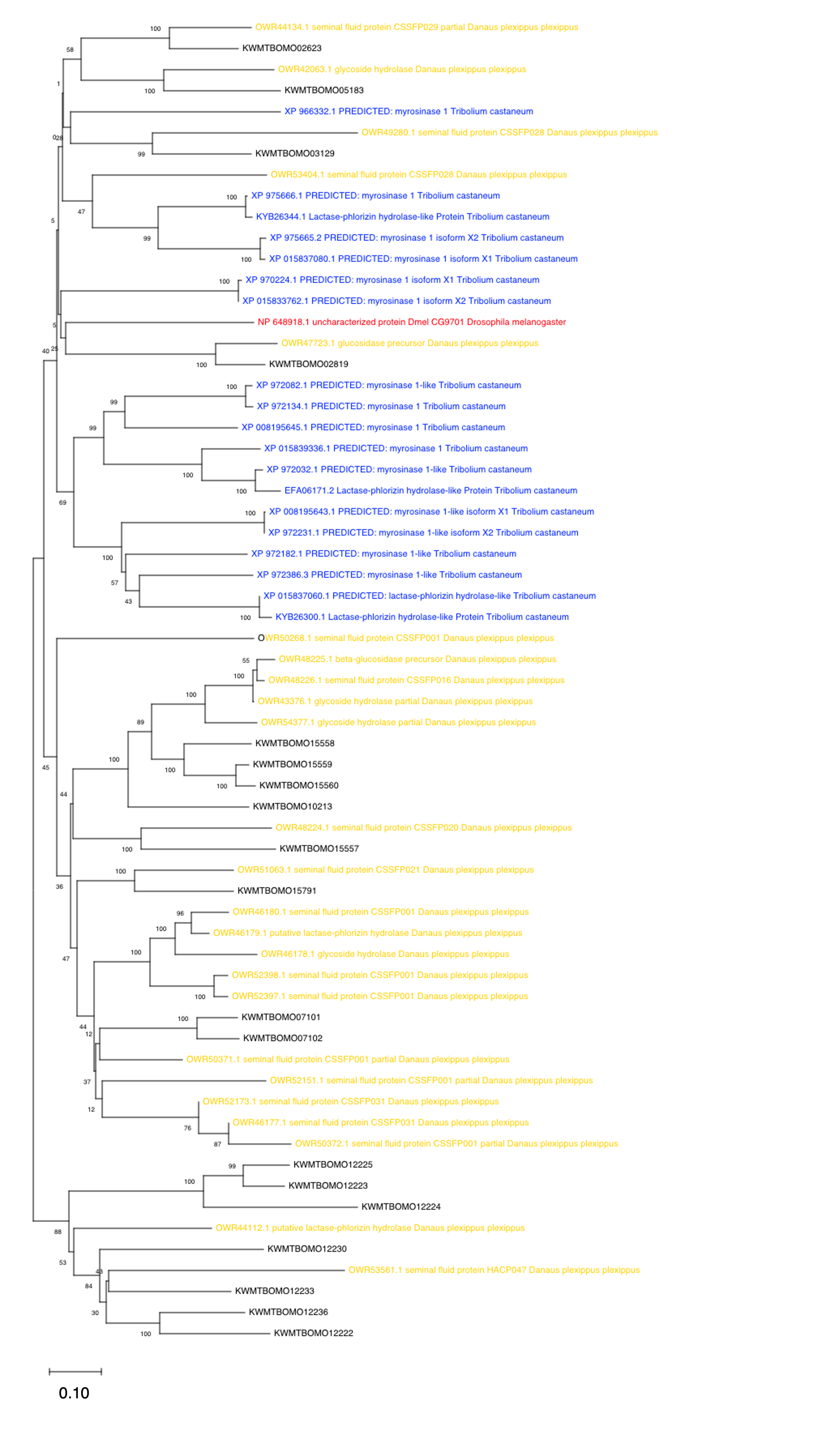

Supplement: S9 Fig — Phylogenetic tree using amino acid sequences of B. mori β-glucosidases. Black: Bombyx mori, red: Drosophila melanogaster, blue: Tribolium castaneum, yellow: Danaus plexippus. Gene models annotated as β-glucosidase were aligned using the MUSCLE program [92], and a phylogenetic tree was built using MEGA X software with the maximum likelihood method [93]. Numbers indicate bootstrap probabilities (%). (TIF) [file pgen.1010912.s015.tif]

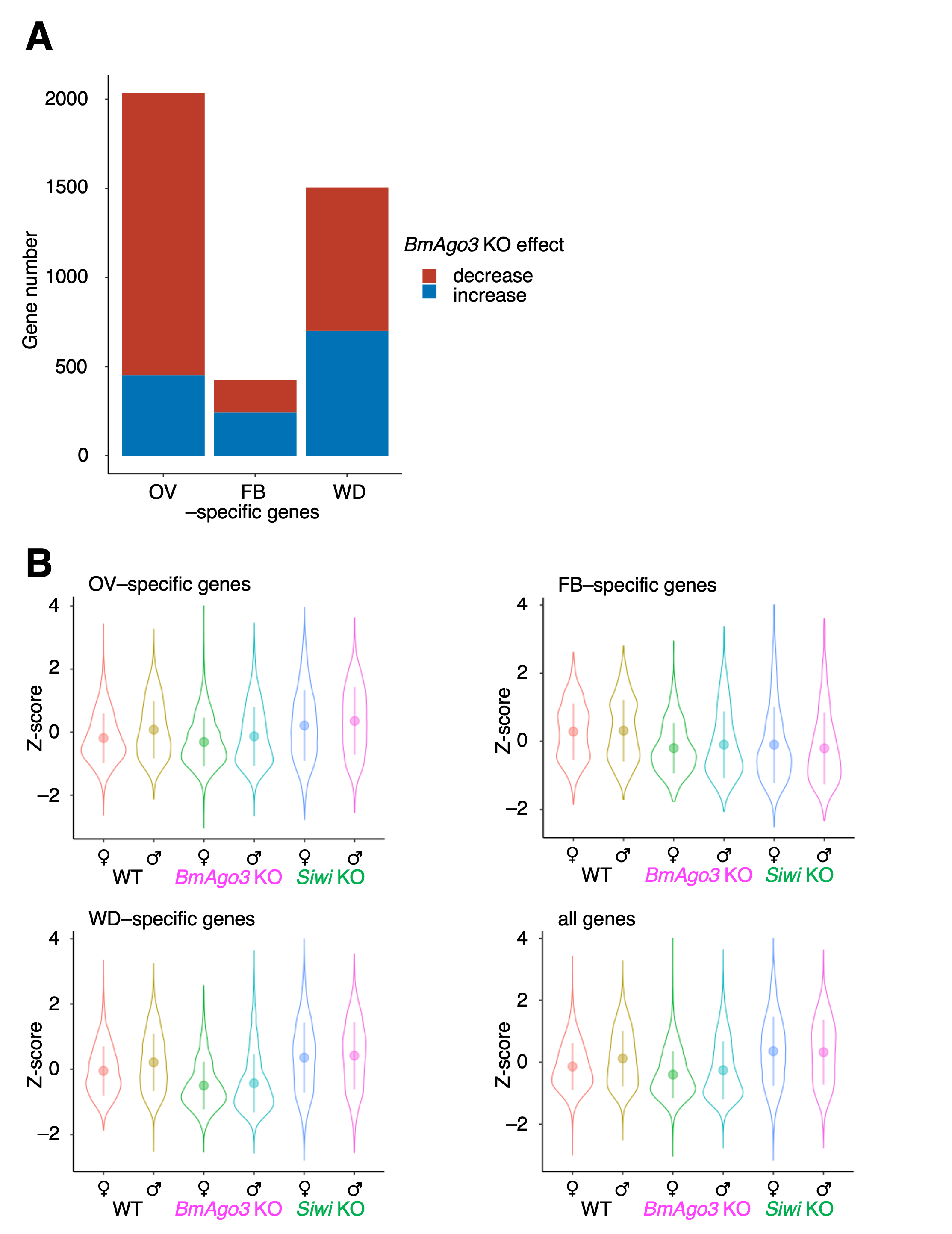

Supplement: S10 Fig — (A) Genes specifically expressed in the ovaries (OV), fat body (FB), and wing discs (WD) in fifth instar larvae were investigated for increased or decreased (1 < log2(Fold Change)) expression in BmAgo3 KO. Each tissue-specific gene was defined as that whose TPM in the WT library of one tissue was more than twice as large as that in the libraries of the other two tissues. (B) Violin plots of tissue-specific gene expression patterns in the whole body of third instar larvae. (TIF) [file pgen.1010912.s016.tif]

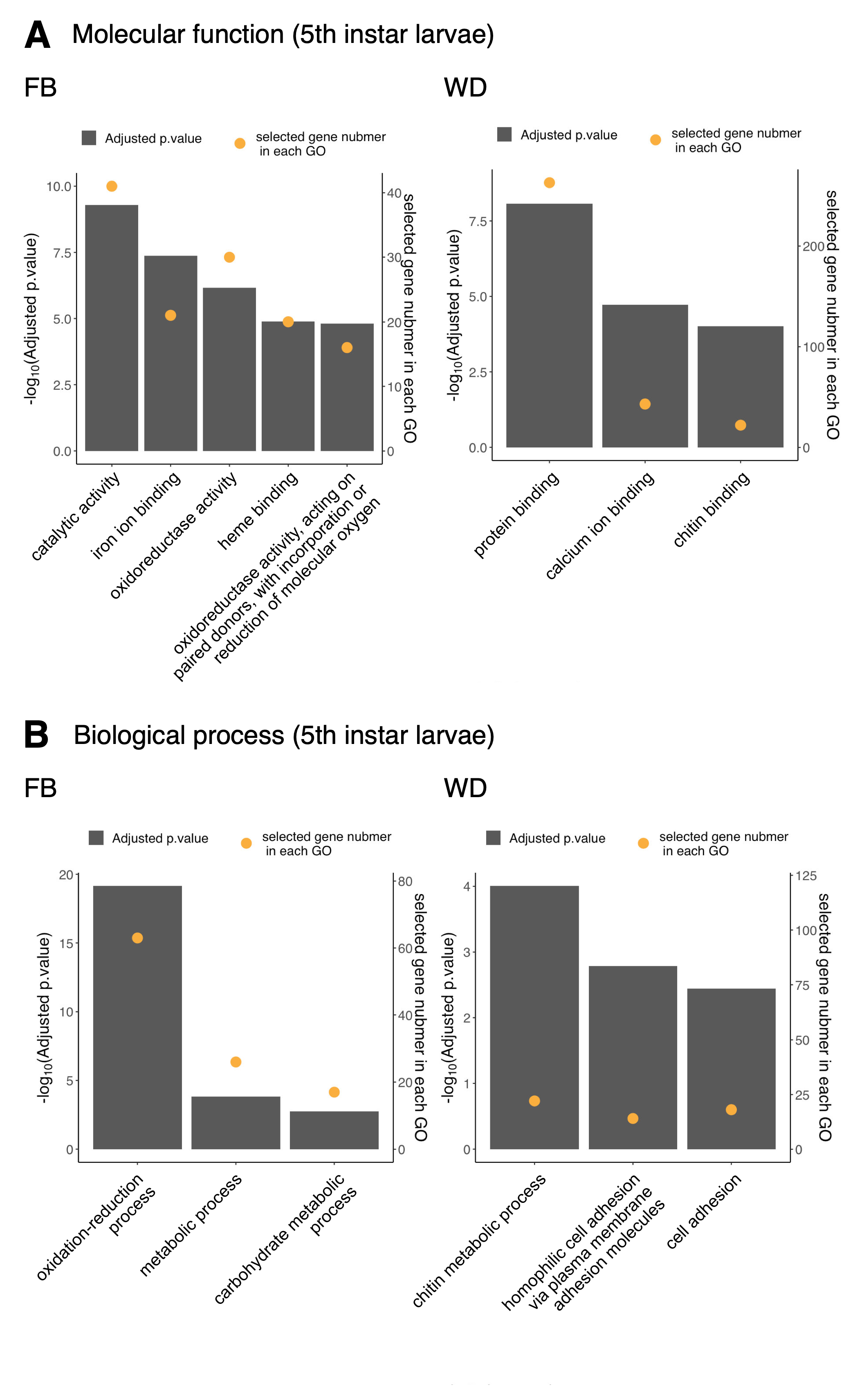

Supplement: S11 Fig — (A and B) GO analysis for molecular function (A) and biological process (B) in the fat body (FB) and wing discs (WD). Bonferroni-adjusted p values (–log10) and selected gene numbers in each GO are shown by gray bars and yellow circles, respectively. (TIF) [file pgen.1010912.s017.tif]
